# Toxic metal mixtures in private well water and increased risk for preterm birth in North Carolina

**DOI:** 10.1186/s12940-023-01021-7

**Published:** 2023-10-16

**Authors:** Lauren A. Eaves, Alexander P. Keil, Anne Marie Jukic, Radhika Dhingra, Jada L. Brooks, Tracy A. Manuck, Julia E. Rager, Rebecca C. Fry

**Affiliations:** 1 Department of Environmental Sciences & Engineering, Gillings School of Global Public Health, University of North Carolina at Chapel Hill, 166A Rosenau Hall, CB #7431, Chapel Hill, NC 27599 USA; 2 Institute for Environmental Health Solutions, Gillings School of Global Public Health, University of North Carolina at Chapel Hill, Chapel Hill, NC USA; 3 Department of Epidemiology, Gillings School of Global Public Health, University of North Carolina at Chapel Hill, Chapel Hill, NC USA; 4 Epidemiology Branch, National Institute of Environmental Health Sciences, Research Triangle Park, Durham, NC USA; 5 Brody School of Medicine, East Carolina University, Greenville, NC USA; 6 School of Nursing, University of North Carolina at Chapel Hill, Chapel Hill, NC USA; 7 Division of Maternal Fetal Medicine, Department of Obstetrics and Gynecology, School of Medicine, University of North Carolina, Chapel Hill, NC USA; 8 Curriculum in Toxicology and Environmental Medicine, University of North Carolina at Chapel Hill, Chapel Hill, NC USA; 9 Department of Pediatrics, School of Medicine, University of North Carolina, Chapel Hill, NC USA

**Keywords:** Metals, Mixtures, Preterm birth, Drinking water, Private well

## Abstract

**Background:**

Prenatal exposure to metals in private well water may increase the risk of preterm birth (PTB) (delivery < 37 weeks’ gestation). In this study, we estimated associations between arsenic, manganese, lead, cadmium, chromium, copper, and zinc concentrations in private well water and PTB incidence in North Carolina (NC).

**Methods:**

Birth certificates from 2003–2015 (*n* = 1,329,071) were obtained and pregnancies were assigned exposure using the mean concentration and the percentage of tests above the maximum contaminant level (MCL) for the census tract of each individuals’ residence at the time of delivery using the NCWELL database (117,960 well water tests from 1998–2019). We evaluated associations between single metals and PTB using adjusted logistic regression models. Metals mixtures were assessed using quantile-based g-computation.

**Results:**

Compared with those in other census tracts, individuals residing in tracts where > 25% of tests exceeded the MCL for lead (aOR 1.10, 95%CI 1.02,1.18) or cadmium (aOR 1.11, 95% CI 1.00,1.23) had an increased odds of PTB. Conversely, those residing in areas with > 25% MCL for zinc (aOR 0.77 (95% CI: 0.56,1.02) and copper (aOR 0.53 (95% CI: 0.13,1.34)) had a reduced odds of PTB. A quartile increase in the concentrations of a mixture of lead, cadmium, and chromium was associated with a small increased odds for PTB (aOR 1.02, 95% CI 1.01, 1.03). This metal mixture effect was most pronounced among American Indian individuals (aOR per quartile increase in all metals: 1.19 (95% CI 1.06,1.34)).

**Conclusions:**

In a large study population of over one million births, lead and cadmium were found to increase the risk of PTB individually and in a mixture, with additional mixtures-related impacts estimated from co-exposure with chromium. This study highlights critical racial and ethnic health disparities in relation to private well water thereby emphasizing the urgent need for improved private well water quality to protect vulnerable populations.

**Supplementary Information:**

The online version contains supplementary material available at 10.1186/s12940-023-01021-7.

## Background

Preterm birth (PTB), defined as delivery prior to 37 completed weeks of gestation, affects 11.1% of births worldwide and is a leading cause of neonatal mortality, making it a pressing global public health challenge [1]. Though its etiology is complex and multifactorial, PTB does have multiple known risk factors including maternal infection, medical co-morbidities, psychosocial stress, poverty, and smoking during pregnancy [2]. Environmental chemicals remain underexamined despite their potential association with PTB risk [2, 3]. Metals, both essential and toxic, are of particular interest for investigation given high exposure levels among reproductive-aged women [4]. Essential metals, including chromium, copper, manganese, and zinc, can be toxic at high concentrations and prior studies have found varying associations with PTB [5–8]. For instance, in some cases, essential metals have been shown to mitigate the effect of toxic metals whereas in others they have been found to accentuate risk [5–8]. Further, despite evidence linking toxic metals, such as lead, with PTB, the hazards posed by other toxic metals/metalloids (e.g., arsenic and cadmium) and their mixtures are less known [6, 8]. Note that while arsenic is technically a metalloid, for ease of reading it will henceforth be referred to as part of the collective term "metals".  

A major source of exposure to metals in the United States (US) is private well water, which serves as the primary drinking water source for approximately 42.5 million Americans (13% of the population) [9]. North Carolina (NC), has the largest population of any state relying on private well water (2.4 million people) [9–11]. The US Environmental Protection Agency (EPA) regulates public drinking water systems under the Safe Drinking Water Act, enforcing standards for arsenic, cadmium, lead, and manganese, among other contaminants [12]. In contrast, private wells are not federally regulated, and water quality stewardship falls on the individual well owner. Consequently, private wells are vulnerable to metal contamination and high arsenic, lead and manganese concentrations have been reported in private well water in NC and elsewhere [13–15]. Well water contamination translates into higher body burdens of metals for well water users compared to public water system users, as has been documented for lead and arsenic [16, 17]. While exposure to metals via private well water has been linked to birth defects, infant mortality and cancer [18–21], few studies have examined the relationship with PTB.

Disparities in rates of PTB by race and ethnicity throughout the US are stark [22]. NC shares a similar pattern: between 2019–2021, the highest prevalence of PTB was among Black individuals (14.6%), followed by American Indian individuals (11.1%), compared to the lowest prevalence among Asian/Pacific Islander individuals and non-Hispanic White individuals, 8.5% and 9.6%, respectively [23]. Recently, there has been a growing recognition of the unequal burden of exposure to environmental chemicals and its potential role in maternal health disparities [24, 25]. When examining upstream forces of environmental health disparities, it is critical to consider that different historical and current forces shape the environmental “riskscape” to which different minority groups are exposed [24, 26]. This is especially pertinent to consider when evaluating private well water-based exposure in NC, as structural environmental racism has led to poor and minority communities being more likely to rely on private well water through the practice of municipal underbounding. With this practice, municipal borders engulf poor and minority communities without expanding services such as water and sewer lines [27–29]. Disproportionate environmental exposures among racial and ethnic groups within the US interact with other complex structural factors including systemic racism, poverty, poor housing, stressful life events and discrimination within the healthcare system; factors that can lead to inflammation, oxidative stress, and a biological phenomenon termed weathering [30–37]. Environmental chemicals can potentially overpower the body's natural detoxification mechanisms, or enhance the biological pathways linking pollutants to adverse effects. They may achieve this by influencing similar mechanisms as the pollutants themselves, such as through epigenetic modifications or disrupting endocrine functions [38, 39]. Thus, social disparities that modify pollutant-outcome associations are increasingly being recognized in environmental health [24, 26, 40, 41]. In a racially stratified society such as the US, self-identified race and ethnicity is often used an imperfect proxy for aforementioned social stressors [34].

In the present study, we set out to test three connected hypotheses to address gaps in the literature: (1) that exposure to toxic metals via private well water would increase the risk of PTB and that exposure to essential metals would reduce the risk of PTB; (2) that exposure to a mixture of both toxic and essential metals would increase the risk of PTB risk, with the essential metals counteracting some of the toxic effects; and (3) that there would be differences in the metal mixture-PTB association by race and ethnicity.

## Methods

### Overview of study design

We constructed the NC-BIRTH cohort from birth certificates of live births in NC between 2003 and 2015. Each birth certificate record was assigned exposure to private well water metal levels based on maternal residence at delivery and either (1) the census tract level mean concentration of the metal or (2) the percentage of exceedances of EPA standard for that metal in well water tests in that census tract.

### Private well water metals database

The NCWELL database was used to generate census tract level mean concentrations of the seven metals of interest: inorganic arsenic (iAs), cadmium (Cd), chromium (Cr), copper (Cu), lead (Pb), manganese (Mn) and zinc (Zn) [13]. We also planned to include mercury in the analysis; however, the number of samples above the limit of reporting (LOR) in the NCWELL database was too low to impute (see further details on imputation below). Herein, arsenic, cadmium and lead were termed “toxic” as there are no known benefits to exposure [42–44]. In contrast, chromium, copper, manganese and zinc are termed “essential” metals as there are benefits to exposure to these metals within safe ranges [45–48]. Note that these distinctions were primarily for interpretation of results and did not guide the analysis.

In brief, the NCWELL database consists of *n* = 117,960 geocoded well water tests analyzed at the NC Department of Health and Human Services, Division of Public Health, State Laboratory of Public Health, between October 19, 1998, and May 20, 2019. The database includes test reports from all 100 counties and 89% of census tracts in NC. Detailed methods for collecting the well water test reports, data cleaning and organization, and state-level and county-level descriptive statistics are described elsewhere [13]. Of note, limited temporal variability over the twenty years of data collection was noted [13].

### Imputation of metal concentrations missing or below the limit of reporting

In a well water test report, either a detected concentration of a metal was listed, or the concentration was listed as below the LOR. The number of non-missing, missing, and below the LOR tests for each metal are detailed in Table S1. The multiple imputation framework was used to account for the missingness of a particular metal’s concentration, either due to lack of measurement in a given test or being below the LOR. For each metal, a left-censored log-linear regression (Tobit regression) was used to model the natural log values of each metal, given the natural log values of every other metal and the county (using indicator variable coding) in which each measurement took place. For measurements below the LOR, the LOR was used as the left censoring value. For missing measurements due to the test not measuring a given metal, the left censoring value was the maximum observed concentration of that metal. The county was included in the model to allow for spatial structure in the measurements. County was the smallest geographic unit at which there were tests within each unit (i.e., some census tracts had no tests recorded). Because cadmium was frequently missing, for statistical efficiency, data from all counties with values (i.e., no non-missing) were used for imputation. Out-of-sample prediction accuracy was assessed during model selection by splitting our sample into two equal proportions, which gave us a final imputation model that included the county and 15 well water measurements (including the seven metals of interest (iAs, Cd, Cr, Cu, Pb, Mn, Zn) as well as calcium (Ca), chlorine (Cl), iron (Fe), magnesium (Mg), sodium (Na), sulfate, total alkalinity, total hardness).

This Tobit model was used within the multivariate imputation through the chained equations (MICE) framework. To detail, first, random values were assigned for all missing metals. Next, for each metal, the model outlined above was fit to the observations with non-missing values. The parameters of that fitted model were re-sampled according to a normal distribution with mean given by the maximum likelihood estimate and standard deviation given by the estimated standard error. Then, the missing values of the metal were imputed according to the expected values given by the fitted model and the re-sampled parameter values. This cycle was repeated over 30 iterations so that the imputations converged and were stable across sequential iterations (checked via trace plots of imputation means and standard deviations) and imputed values from the 30th iteration were kept for subsequent analyses. The imputed data contained values for all metals of interest for every well measurement in the original dataset. Imputation was performed using the “mice” package in R [46, 47] and the Tobit regression routine is available as part of the “qgcomp” package [48, 49].

### Birth certificate data

Birth certificate data were obtained from the NC Birth Defects Monitoring Program (NCBDMP). The NCBDMP is an active, population-based surveillance system operated by the State Center for Health Statistics that collects information about all birth defect cases among NC resident infants [49]. The NCBDMP also collects birth certificate records of all live births in NC, which was the basis of the NC-BIRTH cohort used in this study. Obstetric estimates of gestational age were used for gestational age for this study [50]. GPS-based latitude and longitude of maternal residence at delivery were recorded for most birth certificates. Births with missing GPS information on latitude and longitude were geocoded from maternal residence addresses using ArcGIS (ESRI, Redlands, California). The latitude and longitude coordinates for each birth were then matched to census tracts using the R “tigris” package [51].

We included data on all NC resident live births between 2003 and 2015 (*n* = 1,600,409) (Figure S1). A total of 758 records were removed for having non-NC addresses or inaccurate geographical information. Further, *n* = 104,128 records were removed as they were the birth of multiples or anomalous singletons. These were removed as they have a different etiology of PTB than non-anomalous singletons, the focus of the study. In addition, births reported at gestational ages less than 20 weeks or at or greater than 44 weeks were removed, as reporting of births less than 20 weeks is likely variable and over 44 weeks is likely a clerical error due to current obstetric practices. Lastly, *n* = 164,124 records had a maternal residence at delivery in a census tract with no well water tests; therefore, they could not be assigned exposure and were thus removed. Thus, the final cohort consisted of *n* = 1,329,071 non-anomalous singleton live births.

### Covariate selection

Confounders were selected a priori using a directed acyclic graph (DAG) approach and analyzed utilizing Dagitty (v3.0) [52, 53]. The DAG used is shown in Figure S2. The minimally sufficient set of covariates to control for confounding was identified as an individual’s age, race and ethnicity, smoking status, and socioeconomic status (education and income) as well as season of conception and co-pollutants. Covariates were coded in the following manner: age (years, using a quadratic term), race and ethnicity (White non-Hispanic, Black non-Hispanic, Hispanic, Asian/Pacific Islander, American Indian, Other/unknown, using categorical disjoint indicator variables), smoking status (smoker, non-smoker, as a binary variable), education (less than high school, completed high school, more than high school, as an ordinal categorical variable), tract level income (percentage of residents in a census tract below the poverty line, using quartiles as an ordinal categorical variable), season of conception (winter, spring, summer, fall, as disjoint indicator variables), and co-pollutants (average of the nitrates and nitrites concentration as less than or equal to the 50^th^ percentile, above the 50^th^ percentile and less than or equal to the 90^th^ percentile, or above the 90^th^ percentile, as an ordinal categorical variable). Further details regarding justification for and generation of covariate variables are provided in Additional File 1.

### Single metals modeling

Crude and adjusted (for confounders listed above) logistic regression models were fit to generate odds ratios (OR) and 95% confidence intervals for each of the seven individual metals of interest. Models were fit in R (v4.0.2) using the *glm* function. The primary outcome was PTB (< 37 weeks’ gestation). Secondary outcomes of interest included very PTB (< 32 weeks’ gestation) and extremely PTB (< 28 weeks’ gestation). Using the imputed data described above, for each metal the census tract level mean concentration as well as the percentage of tests exceeding the EPA MCL (or other regulatory standard) were calculated. All standards utilized are listed in Table S1. We note that MCLs are not health-based standards but are set as close as is deemed feasible given treatment and detection technologies to the public health goal of Maximum Contaminant Level Goal (MCLG). The MCLG is the level at which no known adverse effects are expected to occur, including reproductive outcomes such as preterm birth, based on the available epidemiologic and toxicologic evidence. For reference, Table S1 also lists the MCLGs.

Two parameterizations were used to model exposure: one based on the tract level mean concentration and one based on the proportion of tests exceeding the EPA regulatory standard in a tract. For the first, tract level mean metal concentrations were coded as less than or equal to the 50^th^ percentile, above the 50^th^ percentile and less than or equal to the 90^th^ percentile, or above the 90^th^ percentile, generating an ordinal categorical variable. This coding scheme was decided by balancing three competing factors: (1) accurately capturing the functional form (evaluated using quadratic spline plots), (2) generating a reasonable exposure contrast with the constraints of right-skewed data (given a high percentage of values below the LOR), and (3) not relying on extremes of the exposure distribution with lower sample size. For the second approach, binary variables were generated to compare census tracts in which over 25% of well water tests for a given metal exceeded EPA regulatory standard, to those in which less than 25% of tests exceeded the EPA regulatory standard.

### Metal mixtures modeling

To estimate the effect of exposure to metal mixtures via private well water on PTB, we utilized quantile-based g-computation, accessible via the “qgcomp” R package [54, 55]. Quantile-based g-computation estimates an overall mixture effect, defined as the effect of simultaneously increasing all metal concentrations by one quartile, as well as weights for each component exposure, corresponding to its proportion of the negative or positive “partial effect.” Using the *qgcomp.noboot* function, we fit crude and adjusted logistic regression models assessing the effect of all metals on PTB, very PTB, and extremely PTB.

We used sample splitting to estimate partial effects. In this process, the sample is split into a training (30%) and a validation (70%) set with random allocation. In the training dataset, a quantile-based g-computation model was fit to assess whether a metal was considered to have a positive or negative weight. Then, utilizing the validation set, quantile-based g-computation is used to estimate the effect of two distinct metal mixtures while adjusting for other metals: metals found in the training set to have a positive (or negative) effect in the metal mixture. Further details regarding the metal mixtures modeling are provided in Additional File 1.

### Effect measure modification by maternal race and ethnicity

Adjusted ORs (with 95%CI) were estimated within strata of race and ethnicity groups using the “qgcompint” package (v0.7.0) [56], an extension of the qgcomp package. The stratum-specific estimates can be interpreted as the effect of increasing all metal concentrations by one quartile within the index race and ethnicity group.

### Sensitivity analysis

To refine exposure assessment, we repeated the analysis on two subsets of individuals in this cohort more likely to be using private well water as their primary drinking water source, leveraging a dataset of the number of predicted private well users in a census tract based on Johnson et al. 2019 [57]. Specifically, one subset was restricted to only birth certificate records where residence at delivery was in a census tract with 50% or more predicted well water users (*n* = 202,897, 15.3% of the study population), and the second was restricted to census tracts with 25% or more predicted well water users (*n* = 445,002, 33.5%). Further details regarding the sensitivity analysis are provided in Additional File 1.

## Results

### Study population characteristics

The study population included 1,329,071 singleton, non-anomalous (i.e., without congenital defects or aneuploidy) live births in NC between 2003 and 2015 (Table 1). Among these, 124,227 (9.4%) were born preterm, which included 9,495 (0.7%) very PTBs (≥ 28 weeks’ gestation and < 32 weeks’ gestation) and 7,718 (0.6%) extremely PTBs (< 28 weeks’ gestation). PTB prevalence was greater for male infants, individuals who identified as non-Hispanic Black, individuals with less than a high school education, and individuals who smoked during pregnancy. Education and smoking status had the greatest proportion of missing data, with both variables having 8.9% missing.

**Table 1:** Summary of sociodemographic and clinical characteristics of the study population

	**All births**	**Term birth ≥ 37 weeks’ gestation**	**Preterm birth < 37 weeks’ gestation**
	**N (%)**	**N (%)**	**N (%)**
	1,329,071	1,368,968	124,227
**Fetal sex**			
*Male*	675,994 (50.9)	617,728 (50.7)	58,266 (53.0)
*Female*	653,074 (49.1)	601,443 (49.3)	51,631 (47.0)
*Missing*	3	1	2
**Race/ethnicity (of birthing parent)**			
*White non-Hispanic*	773,537 (58.2)	715,722 (58.7)	57,815 (52.6)
*Black non-Hispanic*	292,088 (22.0)	258,455 (21.2)	33,633 (30.6)
*Hispanic*	200,411 (15.1)	186,729 (15.3)	13,682 (12.4)
*Asian/Pacific Islander*	41,364 (3.1)	38,595 (3.2)	2769 (2.5)
*American Indian*	19,286 (1.5)	17,484 (1.4)	1802 (1.6)
*Other/unknown*	2385 (0.2)	2187 (0.2)	198 (0.2)
**Age**			
< *20*	134,939 (10.2)	122,173 (10.0)	12,766 (11.6)
< *30,* > = *20*	711,355 (53.5)	654,089 (53.7)	57,266 (52.1)
< *40,* > = *30*	453,069 (34.1)	416,445 (34.2)	36,624 (33.3)
> = *40*	29,690 (2.2)	26,451 (2.2)	3239 (2.9)
*Missing*	23	19	4
**Education**			
*Less than high school*	249,935 (20.4)	226,962 (20.2)	22,973 (22.7)
*Completed high school*	319,452 (26.1)	290,188 (25.9)	29,264 (28.9)
*More than high school*	654,490 (53.5)	605,434 (53.9)	49,056 (48.4)
*Missing*	118,089	108,399	9690
**Smoking during pregnancy**			
*Non-smoker*	1,086,801 (88.9)	1,001,059 (89.2)	85,742 (84.8)
*Smoker*	136,343 (11.1)	120,916 (10.8)	15,427 (15.2)
*Missing*	118,824	109,012	9812
**Season of conception**			
*Winter (Dec, Jan, Feb)*	336,098 (25.3)	308,664 (25.3)	27,434 (25.0)
*Spring (Mar, Apr, May)*	329,472 (24.8)	301,626 (24.7)	27,846 (25.3)
*Summer (June, Jul, Aug)*	324,489 (24.4)	297,646 (24.4)	26,843 (24.4)
*Fall (Sept, Oct, Nov)*	339,012 (25.5)	311,236 (25.5)	27,776 (25.3)
**Delivery**			
*Vaginal*	947,615 (71.3)	878,675 (72.1)	68,940 (62.7)
*Caesarean*	381,239 (28.7)	340,309 (27.9)	40,930 (37.3)
*Missing*	250	214	36
**Preterm birth severity subtypes**			
*Extreme preterm birth (*< *28 weeks)*	7718 (0.6)	n/a	7718 (7.0)
*Very preterm birth (28 to* < *32 weeks)*	9495 (0.7)	n/a	9495 (8.6)
*Moderate to late preterm birth (32 to* < *37 weeks)*	92,686 (7.0)	n/a	92,686 (84.3)

The NC-BIRTH study population includes singleton, non-anomalous live births between 2003 to 2015 in NC. Specifically, it includes live births in NC of individuals with known date of last menstrual period (LMP) between August 14, 2002 and February 19, 2013 (i.e. gestational age > 20 weeks by January 1, 2003 and < 45 weeks by December 31, 2013)

### Effects of individual metals in private wells on the odds of PTB

#### Metals associated with an increased risk of PTB

**Fig. 1:**
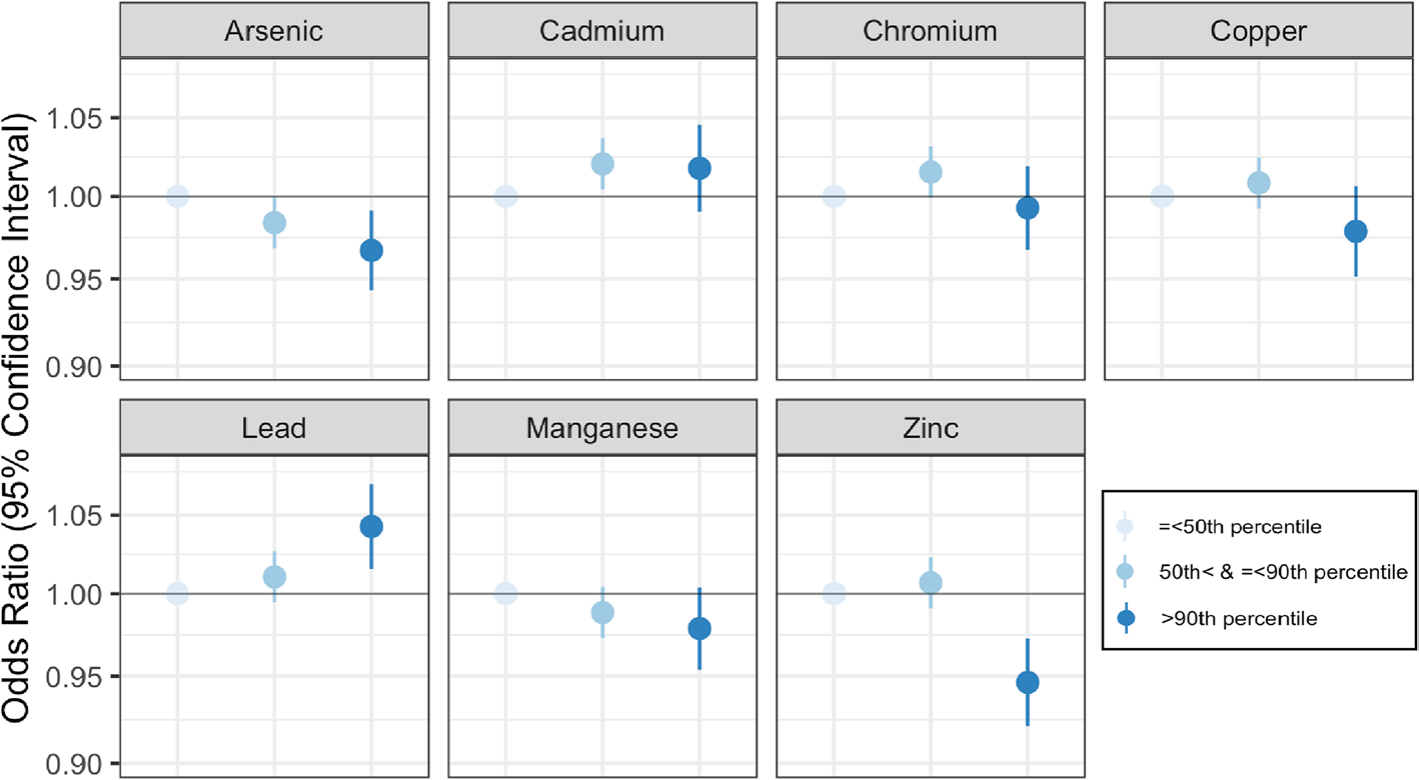
Forest plots of aORs for PTB based on tract-level metal concentrations in private wells. Models were fit comparing individuals in tracts with low (mean tract-level metal concentration =  < 50^th^ percentile of state-wide metal concentration), medium (mean tract-level metal concentration > 50^th^ and < 90th percentile of state-wide metal concentration) and high (mean tract-level metal concentration >  = 90th percentile of state-wide metal concentration). Models were adjusted for smoking, age, race/ethnicity, education, season of conception, tract-level poverty, tract-level nitrates and nitrites

**Fig. 2:**
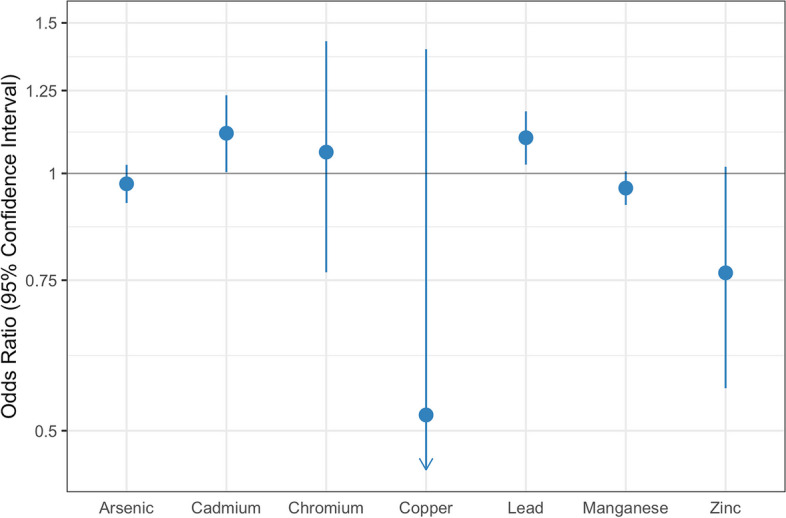
Forest plots of aORs for PTB based on tract-level exceedances of standards in private wells. Models were fit comparing individuals in tracts in which at or over 25% of well water tests reported concentrations above the EPA MCL. Models were adjusted for smoking, age, race/ethnicity, education, season of conception, tract-level poverty, tract-level nitrates and nitrites

We found that cadmium and lead were individually associated with an increased risk of PTB and that chromium may also be a contributing factor. Compared to individuals residing in census tracts with cadmium below the 50^th^ percentile, individuals residing in tracts with cadmium between the 50^th^ and 90^th^ percentile or above the 90^th^ percentile had a slight increase in adjusted odds of PTB (aOR: 1.02 (95% CI: 1.00,1.04), 1.02 (95% CI: 0.99,1.05), respectively) (Table 2, Fig. 1). The effect size was more substantial when evaluating the percentage of tests exceeding the standard as the exposure. Compared to individuals in tracts with less than 25% of tests exceeding the MCL for cadmium (5 ppb), individuals residing in census tracts where 25% or more of the tests exceeded the MCL had 11% higher adjusted odds of PTB (aOR 1.11 (95% CI: 1.00,1.23) (Table 2, Fig. 2).

**Table 2:** Crude and adjusted ORs for PTB (< 37 weeks’ gestation) based on tract-level metal concentrations in private wells

Metal	**ppb**	**Non-cases**	**Cases**	**Crude OR (95% CI)**	Adjusted* OR (95% CI)
**Arsenic**					
< = 50^th^ perc	0.220	609,926	55,433	1.00 (ref.)	1.00 (ref.)
> 50^th^ perc to < 90th perc		487,868	43,709	0.99 (0.97,1.00)	0.98 (0.97,1.00)
> = 90th perc	2.838	121,378	10,757	0.98 (0.95,1.00)	0.97 (0.94,0.99)
tract with < 25% of tests > = EPA standard	Standard=10	1,197,886	21,286	1.00 (ref.)	1.00 (ref.)
tract with > = 25% of tests > = EPA standard		107,999	1900	0.99 (0.94,1.04)	0.97 (0.92,1.02)
**Cadmium**					
< = 50^th^ perc	0.007	609,466	55,164	1.00 (ref.)	1.00 (ref.)
> 50^th^ perc to < 90th perc		488,279	43,653	0.99 (0.98,1.00)	1.02 (1.00,1.04)
> = 90th perc	0.127	121,427	11,082	1.01 (0.99,1.03)	1.02 (0.99,1.05)
tract with < 25% of tests > = EPA standard	Standard=5	1,214,353	4819	1.00 (ref.)	1.00 (ref.)
tract with > = 25% of tests > = EPA standard		109,433	466	1.07 (0.97,1.18)	1.11 (1.00,1.23)
**Chromium**					
< = 50^th^ perc	0.579	608,834	55,735	1.00 (ref.)	1.00 (ref.)
> 50^th^ perc to < 90th perc		488,409	43,538	0.97 (0.96,0.99)	1.02 (1.00,1.03)
> = 90th perc	2.872	121,929	10,626	0.95 (0.93,0.97)	0.99 (0.97,1.02)
tract with < 25% of tests > = EPA standard	Standard= 100	1,214,353	435	1.00 (ref.)	1.00 (ref.)
tract with > = 25% of tests > = EPA standard		109,850	49	1.25 (0.92,1.66)	1.06 (0.77,1.43)
**Copper**					
< = 50^th^ perc	21.096	608,590	56,031	1.00 (ref.)	1.00 (ref.)
> 50^th^ perc to < 90th perc		488,791	43,089	0.96 (0.95,0.97)	1.01 (0.99,1.03)
> = 90th perc	141.545	121,791	10,779	0.96 (0.94,0.98)	0.98 (0.95,1.01)
tract with < 25% of tests > = EPA standard	Standard = 1300	1,214,840	4332	1.00 (ref.)	1.00 (ref.)
tract with > = 25% of tests > = EPA standard		109,510	389	1.00 (0.90,1.10)	0.52 (0.13,1.34)
**Lead**					
< = 50^th^ perc	1.433	609,888	54,784	1.00 (ref.)	1.00 (ref.)
> 50^th^ perc to < 90th perc		487,904	43,740	1.00 (0.99,1.01)	1.01 (1.00,1.03)
> = 90th perc	9.104	121,380	11,375	1.04 (1.02,1.06)	1.04 (1.02,1.07)
tract with < 25% of tests > = EPA standard	Standard= 15	1,191,607	27,565	1.00 (ref.)	1.00 (ref.)
tract with > = 25% of tests > = EPA standard		107,257	2642	1.07 (1.02,1.11)	1.10 (1.02,1.18)
**Manganese**					
< = 50^th^ perc	33.626	609,411	55,515	1.00 (ref.)	1.00 (ref.)
> 50^th^ perc to < 90th perc		488,057	43,761	0.98 (0.97,1.00)	0.99 (0.97,1.00)
> = 90th perc	188.923	121,704	10,623	0.96 (0.94,0.98)	0.98 (0.95,1.00)
tract with < 25% of tests > = EPA standard	Standard= 300	1,176,657	42,515	1.00 (ref.)	1.00 (ref.)
tract with > = 25% of tests > = EPA standard		106,221	3678	0.96 (0.93,0.99)	0.96 (0.92,1.01)
**Zinc**					
< = 50^th^ perc	126.764	608,922	55,763	1.00 (ref.)	1.00 (ref.)
> 50^th^ perc to < 90th perc		487,844	43,642	0.98 (0.96,0.99)	1.01 (0.99,1.02)
> = 90th perc	1592.883	122,406	10,494	0.94 (0.92,0.96)	0.95 (0.92,0.97)
tract with < 25% of tests > = EPA standard	Standard= 5000	1,210,117	9055	1.00 (ref.)	1.00 (ref.)
tract with > = 25% of tests > = EPA standard		109,200	699	0.86 (0.79,0.92)	0.77 (0.56,1.02)

Note that for each metal, two models were fit. First, models were fit comparing individuals in tracts with low (mean tract-level metal concentration =  < 50^th^ percentile of state-wide metal concentration), medium (mean tract-level metal concentration > 50^th^ and < 90th percentile of state-wide metal concentration) and high (mean tract-level metal concentration >  = 90th percentile of state-wide metal concentration). Second, models were fit comparing individuals in tracts in which at or over 25% of well water test reported concentrations above the EPA standard. Models were adjusted for smoking, age, race/ethnicity, education, season of conception, tract-level poverty, tract-level nitrates and nitrites

Lead demonstrated an increasing monotonic trend with respect to PTB. As with cadmium and chromium, the effect sizes were generally small. Compared to individuals residing in census tracts with lead below the 50^th^ percentile, those residing in tracts with lead between the 50^th^ and 90^th^ percentile had a 1% increase in the adjusted odds of PTB (aOR 1.01 (95% CI: 1.00,1.03)) and those in tracts with lead above the 90^th^ percentile had a 4% increased adjusted odds of PTB (aOR 1.04 (95% CI: 1.02,1.07) (Table 2, Fig. 1). In addition, compared to individuals in tracts with less than 25% of tests exceeding the MCL for lead (15 ppb), individuals residing in census tracts where 25% or more of tests exceeded the MCL had 1.10 (95% CI: 1.02,1.18) times the adjusted odds of PTB (Table 2, Fig. 2).

Chromium was associated with a small increased risk of PTB, although findings were more imprecise and varied across different exposure parameters. Compared to individuals residing in census tracts with chromium below the 50^th^ percentile, individuals residing in tracts with cadmium between the 50^th^ and 90^th^ percentile had 1.02 times the adjusted odds of PTB (95% CI: 1.00, 1.03) (Table 2, Fig. 1). However, the adjusted odds ratio corresponding to individuals in tracts with chromium above the 90^th^ percentile was 0.99 (95% CI 0.97, 1.02), thus a monotonic dose response was not observed. While the confidence intervals were wide, when evaluating exposure as the percentage of tests that exceeded the MCL, chromium was also associated with an increase in PTB (aOR 1.06 (95% CI: 0.77,1.43) (Table 2, Fig. 2).

In general, for these three metals, evaluations comparing the 50^th^ and 90^th^ percentile exposures yielded small aORs that were close to the null, suggesting that there may be a true small effect at these exposure ranges or that the effect is null. However, assessing exposure based on the percentage of exceedances of the MCL within a tract yielded stronger effect sizes, which suggests that the effect on preterm birth may occur primarily at high exposure levels.

#### Metals associated with a reduction in the risk of PTB

Copper and zinc concentrations in private wells were associated with reduced odds of PTB (Table 2, Fig. 1). Specifically, compared to individuals residing in census tracts with copper or zinc below the 50^th^ percentile, individuals residing in census tracts with copper or zinc above the 90^th^ percentile had 2–5% lower adjusted odds of PTB (copper aOR 0.98 (95% CI: 0.95,1.01), zinc aOR 0.95 (95% CI: 0.92, 0.97)). When assessing residence in tracts with > 25% of tests exceeding the MCL as the exposure, both copper and zinc were associated with substantial reductions in the adjusted odds of PTB (copper aOR 0.53 (95% CI: 0.13,1.34), zinc aOR 0.77 (95% CI: 0.56,1.02)) (Table 2, Fig. 2). However, both of these estimates were accompanied with large confidence intervals.

Arsenic and manganese both demonstrated small monotonic dose response trends with increasing metal concentrations associated with reductions in adjusted odds of PTB (Table 2, Fig. 1). Compared to individuals residing in census tracts with arsenic below the 50^th^ percentile, those in tracts with arsenic above the 90^th^ percentile had 0.97 times the adjusted odds of PTB (95% CI: 0.94,0.99). Similarly, compared to individuals residing in census tracts with manganese below the 50^th^ percentile, those in tracts with manganese above the 90^th^ percentile had 0.98 times the adjusted odds of PTB (95% CI: 0.95,1.00).

### Effect of metal mixtures in private wells on the odds of PTB

Increasing the mean census tract concentration of all metals by one quartile was not notably associated with PTB (aOR: 0.98 (95% CI: 0.94,1.02) (Table 3). Cadmium and lead had the largest positive weights (0.52 and 0.33, respectively), indicating that they contributed the most towards increasing the odds of PTB within the mixture (Table 3). Conversely, zinc and arsenic had the largest negative weights (-0.37 and -0.36, respectively), indicating that they contributed the most towards decreasing the odds of PTB within the mixture.

**Table 3:** Summary of results from quantile-based g-computation modeling

Model	Interpretation	Crude OR (95% CI)	Adjusted* OR (95% CI)	Adjusted weights		Adjusted coefficients	
A) Standard quantile-based g-computation	Increasing all metals by one quartile (ppb)	0.97 (0.96,0.98)	1.00 (0.99,1.02)	Cadmium Lead Chromium Zinc Arsenic Copper Manganese	0.52 0.33 0.15 -0.37 -0.36 -0.20 -0.07	Cadmium Lead Chromium Zinc Arsenic Copper Manganese	0.015 0.009 0.004 -0.009 -0.009 -0.005 -0.002
B) Positive direction partial effects quantile-based g-computation	Increasing all metals that were in the positive direction in the training set by one quartile (ppb)	1.00 (0.99,1.01)	1.02 (1.01,1.03)	Cadmium Lead Chromium	0.65 0.20 0.15	Cadmium Lead Chromium	0.012 0.004 0.003
C) Negative direction partial effects quantile-based g-computation	Increasing all metals that were in the negative direction in the training set by one quartile (ppb)	0.96 (0.95,0.97)	0.99 (0.97,1.00)	Zinc Arsenic Copper Manganese	-0.51 -0.43 -0.05 1.00	Zinc Arsenic Copper Manganese	-0.007 -0.006 -0.001 0.001

Model A includes all metals in the exposure matrix. Model B and C contain metals that were associated in the positive direction and the negative direction, respectively in the training data set in the quantile-based g computation partial effect modelling. The weights, that sum to 1 or -1 for each direction, represent the proportion of each metal’s contribution to the partial effect in the negative (weight < 0) or positive (weight > 0) direction. The adjusted coefficient for each metal represents the independent effect size for that metal. Models were adjusted for smoking, age, race/ethnicity, education, season of conception, tract-level poverty, tract-level nitrates and nitrites

**Fig. 3:**
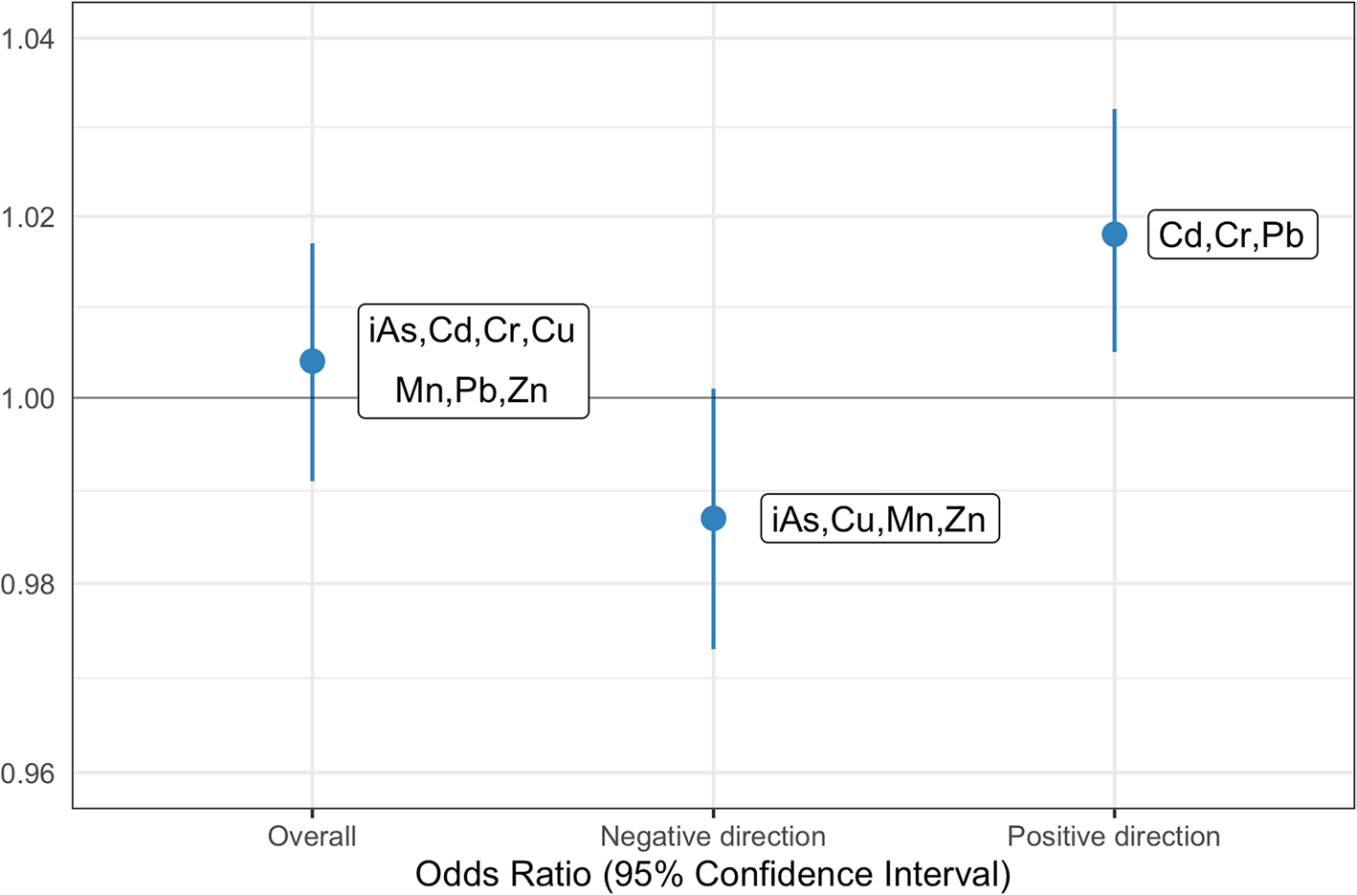
Results from the partial effects quantile-based g-computation modeling. Forest plot of aOR* for PTB associated with increasing all metal concentrations in private wells by one quartile (“Overall”), just the metals that had negative weights in the training set (“Negative direction”), and just the metals that had a positive weights in the training set (“Positive direction”). Models were adjusted for smoking, age, race/ethnicity, education, season of conception, tract-level poverty, tract-level nitrates and nitrites

In the partial effects approach, a more pronounced effect was observed. Specifically, cadmium, lead, and chromium were all identified to be positively associated with PTB in the training set. In the validation dataset, increasing the mean census tract concentration of each of these by one quartile was associated with 1.02 (95% CI: 1.01,1.03) times the adjusted odds of PTB (Table 3, Fig. 3). Cadmium most strongly contributed to this association with a 0.65 weight, followed by lead with a 0.20 weight, mirroring the single metals findings (Table 3). Increasing the mean census tract concentration of manganese, zinc, arsenic, and copper by one quartile (all identified to be negatively associated with PTB in the training set) was associated with 0.99 times the adjusted odds of PTB (95% CI: 0.97,1.00) (Table 3). Manganese within this metal mixture was positively associated with PTB (Table 3). Mixture components that switch direction between the training and validation sets can be interpreted to likely have null partial effects, reinforced by manganese’s small individual coefficient value. These results were robust to different training sets, which is described in further detail in Additional File 1 and Tables S2 and S3.

### Racial and ethnic disparities of the effect of metal mixture exposure on PTB

When the effect of increasing all metals by one quartile was examined within strata of race and ethnicity, the effect was most pronounced among American Indian individuals. Specifically, a one-quartile increase in the mean census tract concentration of all metals was associated with 20% higher adjusted odds of PTB among American Indian individuals (OR: 1.19 (95% CI: 1.06,1.34)) (Table 4). Asian and/or Pacific Islander individuals also had an increased risk of PTB in relation to the metal mixture (aOR: 1.08 (95% CI: 0.99, 1.18)). Black non-Hispanic individuals, Hispanic individuals, and individuals for whom their race was coded in the birth certificate as Other or unknown had stratum specific aORs slightly below the null.

**Table 4:** Metal mixture effects on preterm birth stratified by race and ethnicity

	Adjusted^a^ OR (95% CI) for metal mixture within strata of race and ethnicity
White non-Hispanic	1.00 (0.98,1.02)
Black non-Hispanic	0.98 (0.96,1.00)
Hispanic	0.97 (0.94,1.01)
Asian/Pacific Islander	1.08 (0.99,1.18)
American Indian	1.19 (1.06,1.34)
Other/unknown	0.95 (0.69,1.30)

^a^adjusted for smoking, age, education, season of conception, tract-level poverty, tract-level nitrates and nitrites

### Metals, metal mixtures and very or extremely PTB

Overall, similar point estimate trends were observed for all individual metals when looking at the outcome of very or extremely PTB, albeit most confidence intervals were wide and spanned the null, likely due to the reduced sample size of cases (Table S4). Notably, for lead, compared to individuals in tracts with < 25% of tests exceeding the MCL, individuals residing in census tracts where over 25% of tests exceeded the MCL had 1.46 (95% CI: 1.17,1.81) times the adjusted odds of extremely PTB, mirroring findings from the main PTB analysis (Table S4).

In the mixtures models, increasing all exposures by one quartile did not produce notable associations with very or extremely PTB as outcomes in the overall or partial effects approach (Table S5). Still, similar trends were observed as with the PTB analysis regarding the directionality of individual metals contributing to the overall mixture effects (Table S5).

### Sensitivity analysis

As a sensitivity analysis, the models were run on two subsets of the data: one restricted to only birth certificate records where maternal residence at delivery was in a census tract with 50% or more predicted well water users and one restricted to census tracts with 25% or more predicted well water users. In both subsets of the cohort, we observed similar estimates as in the full analysis. However, in most cases, the confidence intervals were wider, encompassing the null. Results for the sensitivity analysis are described in detail in Additional File 1 and in Tables S6 and S7.

## Discussion

In this study, we evaluated over 1.3 million births in NC to assess the risk of PTB in relation to exposure to a suite of metals (arsenic, cadmium, chromium, copper, manganese, lead, and zinc), both individually and in mixtures. We focused on exposure via private well water, an under-researched source of exposure [27], and employed a novel mixtures methodology to understand the effect of exposure to multiple metals. To our knowledge, this study is among the first to utilize partial effects quantile-based g-computation to test mixture effects of metals on PTB. This approach is beneficial for distinguishing the metals with the strongest influence on the outcome of PTB. We found that private well water levels of lead and cadmium were individually associated with an increased risk of PTB. In contrast, zinc and copper were associated with a reduced risk of PTB. A mixture of cadmium, lead, and chromium was also associated with an increased risk of PTB. In addition, we found that the effect of the overall metal mixture was most pronounced among individuals who identified as American Indian, highlighting the need to center environmental justice in consideration of the consequences of metal exposure via private wells.

Other studies have shown that lead exposure increases the risk of PTB, even at low levels [3]. The present study confirms this relationship and highlights the specific context of private well water exposure. This is critical because private well water users are often more vulnerable to lead contamination than those on public water [11, 15, 16, 27]. While fewer studies have assessed the relationship between cadmium exposure and PTB compared to lead, there is evidence to suggest that prenatal cadmium exposure increases the risk of PTB [58]. This may, in part, be driven by the well-recognized link between cadmium exposure and preeclampsia, the leading etiology of medically-indicated PTB [59, 60]. Interestingly, selenium and zinc may attenuate the effect of cadmium on preeclampsia risk, suggesting a role for dietary interventions and reinforcing the potentially protective role of zinc, as observed in the present study [60, 61].

Our mixtures modeling approach also identified lead and cadmium to be key contributors to the overall mixture effect on PTB. This finding reinforces the value of using a data-adaptive approach to parse out the effect of risk-inducing and risk-reducing components that, when examined together, may mask the toxicity of compounds within a mixture. This knowledge can be used to inform the development of targeted interventions, such as water filtration systems, that reduce toxic contaminants while avoiding reductions in beneficial nutrients. Few studies have evaluated the role of mixtures of metals on PTB risk. One study by Ashrap et al*.*, found that lead was also a dominant driver of toxicity among a toxic metal group, mirroring our findings [6]. In contrast, Kim et al*.* found that only the essential metals, not the toxic or seafood-intake-related metals, were associated with an increased risk of PTB [8]. Inconsistencies across studies may be driven by differences in exposure assessment (i.e., environmental measures versus individual biomarkers), mixtures modeling approaches, and the populations studied.

One unexpected finding was a lower risk of PTB associated with arsenic and manganese well water concentrations in both individual and mixture assessments. This finding is unlikely to be a true effect of arsenic, a known developmental toxicant that has been linked to low birth weight, spontaneous abortion, and other adverse birth outcomes [62, 63]. Manganese is essential at low doses; however, high manganese concentrations in NC well water have been linked to congenital disabilities and infant mortality, again making a true protective effect on PTB risk unlikely [18, 21]. Of note, this analysis excluded anomalous births; therefore, this etiology of PTB would not have been captured in these data. Arsenic and manganese are highly co-occurring in NC; thus, their similar trends with PTB are likely due to their correlation [13, 18]. Furthermore, arsenic has been documented to co-occur with other metals, such as iron and selenium, that were not considered in this study that may represent residual confounding explaining this counterintuitive finding [64, 65].

Lastly, the metal mixture effect was most pronounced among American Indian individuals, which most likely reflects the environmental injustices American Indians have endured for centuries. American Indians – historically forcefully removed from their tribal homelands and relocated to unoccupied lands contaminated and exploited by corporate and government entities – are disproportionately exposed to countless environmental hazards [66, 67]. American Indian populations, including pregnant women, have been documented to have higher than the national average blood and/or urine levels of lead, cadmium, arsenic, and manganese for which private well contamination is a likely contributor [68–73]. In the context of this study, in which we did not directly link individuals with well water exposures, this finding likely reflects variation between racial and ethnic groups in how accurately census tract level average well water concentrations equate to personal exposure. Specifically, given historical and current environmental injustices, including municipal underbounding and relocation to polluted land, American Indian individuals are more likely than other groups to use contaminated well water [27, 29, 67]. Furthermore, given high levels of poverty among American Indian populations in NC (21.2% live below the poverty line compared to 6.7% for non-Hispanic whites [74]), these communities likely face financial barriers to well water quality stewardship and household-level protections in the case of contamination. In a recent NC-based study, American Indian participants were significantly less likely than White participants to have access to a water filter [75]. Furthermore, these populations may rely on support from county health department well water programs, which have been characterized as fractured and under-resourced, particularly in poorer counties [11, 76, 77]. Thus, due to a lack of support for well water users among these communities, American Indians’ personal metal exposure may more directly correlate with local well water concentrations compared to other racial and ethnic groups. Despite evidence of the health effects of toxic metals among American Indians [78–81], few studies have examined metals’ contributions to reproductive health outcomes specifically, especially in the Southeastern US, making our study finding particularly novel. Our findings highlight the need to tackle well water quality concerns among American Indian populations in NC by extending water service lines to peri-urban areas that have experienced municipal underbounding and providing resources for free or low-cost testing and treatment, such as household tabletop water filters [27, 82–84].

While this study is one of the largest to date to assess metal exposure via private drinking wells and PTB, it is not without limitations. First, while our finding that cadmium is associated with an increased risk of PTB is biologically plausible, it should still be interpreted cautiously because air pollution, diet, and smoking, are other major exposure sources for cadmium, and detectable levels of cadmium in the NCWELL database were low. Second, it is important to note that data were constrained to maternal self-report of race and ethnicity among checklist options on the birth certificate. There are numerous ways to measure race and ethnicity, and self-report does not capture experiences of discrimination and racism, a driver of racial health disparities [85]. This may explain, in part, why we did not observe an expected exacerbated metal mixture effect among Black non-Hispanic individuals, despite this community’s baseline risk and likelihood of exposure being higher. Other reasons for this unexpected finding could include the rural–urban residential distribution (which influences likelihood of being on private well water) of Black non-Hispanic individuals in the state and the representativeness of well water testing among different minority groups [86]. Furthermore, “American Indian” as a grouping is a conglomerate term including federal and state-recognized tribes in NC, as well as members of non-recognized tribes, all with unique cultural and economic forces shaping the reproductive health of these individuals [74]. Third, by design, this study used an environmental exposure assessment, a proxy for personal internal dose which includes both actual intake of contaminated drinking water and other sources of exposure including food and air pollution. However, the birth certificates do not include data on maternal drinking water source or use of water filters, likely leading to non-differential exposure misclassification. In this study design, we assumed that the residential address at birth was representative of the address throughout pregnancy, likely also leading to non-differential exposure misclassification. Based on available data, it is not possible to determine the exact source of metals detected in well water (ie. geogenic sources, piping-derived and/or industry contamination, among other potential sources). Future research should investigate whether interventions to reduce well water-based toxic metal exposure impact PTB risk, especially among vulnerable communities, and should aim to identify specific sources of well water metals contamination. Fourth, while the NCWELL database is one of the largest databases of its kind, it has limitations that have been previously documented [13]. Of particular relevance, the lead samples collected are generally flush samples which most likely underestimate true lead exposure [15, 87]. Lastly, the use of a birth certificate cohort allowed us to examine a large population; however, detailed information on each individual was limited therefore we could not differentiate between spontaneous and medically-indicated PTB nor control for potential confounding by prior pregnancy and medical history. The use of birth certificates (live births only) also induces live-birth bias, which may underestimate social disparities resulting from environmental exposures [88].

## Conclusions

In conclusion, we analyzed 1.3 million birth certificates and the NC-WELL database (twenty years of geocoded well water tests in NC) to evaluate the association between well water metal contamination and PTB. We found that concentrations of lead and cadmium in private well water were associated with PTB both in single-exposure and mixture-based analyses and that zinc was associated with a reduction in the risk of PTB. We also documented an enhanced metal mixture effect on increasing the risk of PTB among American Indian individuals, highlighting the need to use an environmental justice lens to address well water quality concerns and maternal/child health disparities in NC.

### Supplementary Information

**Additional file 1. **Supplemental Information for Methods.  Supplemental Information for Results. **Figure S1.**  Flow diagram demonstrating the construction of the retrospective cohort used in the analysis, derived from live births between 2003 to 2015* in North Carolina. **Figure S2.** Directed Acyclic Graph describing relationship between well water metal concentrations, preterm birth, and critical covariates. **Table S1.** The EPA Maximum Contaminant Level Goal (MCLG) and EPA regulatory standard used in the study and well as the number and percentage of well water tests above and below limit of reporting for each metal. **Table S2.** Summary of the results from the 12 repeated cycles of partial effects quantile-based g-computation. **Table S3.** Summary of the results from the 12 repeated cycles of partial effects quantile-based g-computation. **Table S4.** Crude and adjusted odds ratios for the odds of (A) very preterm birth and (B) extremely preterm birth, comparing individuals in tracts with varying concentrations of individual metal concentrations reported in private wells. **Table S5.** Summary of results from quantile-based g-computation modeling for very preterm birth and extremely preterm birth outcomes. **Table S6.** Adjusted odds ratios for the odds of preterm birth, comparing individuals in tracts with varying concentrations of individual metal concentrations reported in private wells. **Table S7.** Summary of results from adjusted quantile-based g-computation modeling among the (A) population residing in tracts with over 25% of residents estimated to be private well water users and (B) population residing in tracts with over 50% of residents estimated to be private well water users.

## Data Availability

Data and code can be found on the UNC-SRP GitHub repository: https://github.com/UNCSRP/Toxic-metal-mixtures-in-private-well-water-and-increased-risk-for-preterm-birth-in-North-Carolina. Geocoded birth certificate records are not publicly available to protect participant privacy. Individual level well data are also not publicly available in order to protect NC residents on private well water, a decision that mirrors the original publication of the NCWELL data [13]. The dataset of the census tract level metal concentrations, based on the imputation described above, is available on Github, as are the analysis scripts used to generate this dataset and the results presented throughout.

## Abbreviations

aOR: Adjusted odds ratio
Cd: Cadmium
Cr: Chromium
Cu: Copper
DAG: Directed acyclic graph
EPA: Environmental Protection Agency
iAs: Inorganic arsenic
LOR: Limit of reporting
MCL: Maximum contaminant level
MICE: Multiple imputation with chained equations
Mn: Manganese
NC: North Carolina
NCBDMP: North Carolina Birth Defects Monitoring Program
Pb: Lead
ppb: Parts per billion
PTB: Preterm birth
US: United States
Zn: Zinc
