# Supplementary material for: Toxic metal mixtures in private well water and increased risk for preterm birth in North Carolina
Source: Environ Health. 2023 Oct 16;22:69. doi: 10.1186/s12940-023-01021-7 (PMC10577978; doi:10.1186/s12940-023-01021-7)
Supplement: Supplementary file 1 — Additional file 1. Supplemental Information for Methods. Supplemental Information for Results. Figure S1. Flow diagram demonstrating the construction of the retrospective cohort used in the analysis, derived from live births between 2003 to 2015* in North Carolina. Figure S2. Directed Acyclic Graph describing relationship between well water metal concentrations, preterm birth, and critical covariates. Table S1. The EPA Maximum Contaminant Level Goal (MCLG) and EPA regulatory standard used in the study and well as the number and percentage of well water tests above and below limit of reporting for each metal. Table S2. Summary of the results from the 12 repeated cycles of partial effects quantile-based g-computation. Table S3. Summary of the results from the 12 repeated cycles of partial effects quantile-based g-computation. Table S4. Crude and adjusted odds ratios for the odds of (A) very preterm birth and (B) extremely preterm birth, comparing individuals in tracts with varying concentrations of individual metal concentrations reported in private wells. Table S5. Summary of results from quantile-based g-computation modeling for very preterm birth and extremely preterm birth outcomes. Table S6. Adjusted odds ratios for the odds of preterm birth, comparing individuals in tracts with varying concentrations of individual metal concentrations reported in private wells. Table S7. Summary of results from adjusted quantile-based g-computation modeling among the (A) population residing in tracts with over 25% of residents estimated to be private well water users and (B) population residing in tracts with over 50% of residents estimated to be private well water users. [file 12940_2023_1021_MOESM1_ESM.docx]

**Toxic metal mixtures in private well water and increased risk for preterm birth in North Carolina**

Lauren A. Eaves^1,2^, Alexander P. Keil^3^, Anne Marie Jukic^4^, Radhika Dhingra^1,5^, Jada L. Brooks^6^, Tracy A. Manuck^2,8^, Julia E. Rager^1,2,8^ and Rebecca C. Fry^1,2,7,9^

1. Department of Environmental Sciences & Engineering, Gillings School of Global Public Health, University of North Carolina at Chapel Hill, Chapel Hill, NC, USA
2. Institute for Environmental Health Solutions, Gillings School of Global Public Health, University of North Carolina at Chapel Hill, Chapel Hill, NC, USA
3. Department of Epidemiology, Gillings School of Global Public Health, University of North Carolina at Chapel Hill, Chapel Hill, NC, USA
4. Epidemiology Branch, National Institute of Environmental Health Sciences, Research Triangle Park, Durham, NC, USA
5. Brody School of Medicine, East Carolina University, Greenville, NC, USA
6. School of Nursing, University of North Carolina at Chapel Hill, Chapel Hill, NC, USA
7. Curriculum in Toxicology and Environmental Medicine, University of North Carolina at Chapel Hill, Chapel Hill, NC, USA
8. Department of Obstetrics and Gynecology, Division of Maternal Fetal Medicine, School of Medicine, University of North Carolina, Chapel Hill, NC, USA
9. Department of Pediatrics, School of Medicine, University of North Carolina, Chapel Hill, NC, USA

**Supplemental Information for Methods**

***Covariate selection***. Age (given in years), and smoking status during pregnancy (yes/no) are recorded on the birth certificate and are well known risk factors for PTB [(1,2)](https://sciwheel.com/work/citation?ids=14433276,8482414&pre=&pre=&suf=&suf=&sa=0,0&dbf=0&dbf=0). To capture SES, we used income and education. SES is a well-known risk factor for PTB and influences the likelihood of exposure to metals via well water due to the cost of testing and treatment [(3)](https://sciwheel.com/work/citation?ids=4216093&pre=&suf=&sa=0&dbf=0). Education was reported on the birth certificate (less than high school, completed high school, more than high school, coded as an ordinal categorical variable). Tract level income was used as a proxy for individual income, which was not captured on the birth certificate. Instead, the percentage of residents in a census tract below the poverty line, averaging measures from the American Communities Survey in 2010 and 2015 (coded using quartiles as an ordinal categorical variable) was used. The R package “tidycensus” (v1.2.1) was used to download these data [(4)](https://sciwheel.com/work/citation?ids=11789041&pre=&suf=&sa=0&dbf=0).

The justification for the inclusion of race and ethnicity as a confounder (and potential modifier) in this analysis is detailed extensively in the Introduction. Race and ethnicity is captured on the birth certificate; however, the methods for collection changed over the course of the study period. In NC, the 2003 version of the birth certificate was implemented in 2010, with 2011 being the first year where all records were completed using the 2003 form. Before the 2003 revision, maternal race and ethnicity were written in by the parent in a free-text field and then were subsequently coded [(5)](https://sciwheel.com/work/citation?ids=7058715&pre=&suf=&sa=0&dbf=0). In the 2003 revision, there are checkboxes provided for (1) identifying as Hispanic or not, and (2) racial identification categories [(6)](https://sciwheel.com/work/citation?ids=14433251&pre=&suf=&sa=0&dbf=0). The NC Birth Defects Monitoring Program (NCBDMP) reports data to the Centers for Disease Control and Prevention (CDC) for the National Birth Defects Prevention Network surveillance reports and therefore follows the CDC standards, which require the classifications used in this study (White non-Hispanic, Black non-Hispanic, Hispanic, Asian/Pacific Islander, American Indian, Other/unknown). Note that in the US context, Hispanic or non-Hispanic refers to one’s ethnicity, while Black, White, Asian and/or Pacific Islander and American Indian are racial categories [(7)](https://sciwheel.com/work/citation?ids=11552465&pre=&suf=&sa=0&dbf=0). Generally, ethnicity refers to one’s cultural identity and race more to broad socially-constructed categories based on ancestral origins; thus, these are connected but not identical constructs [(7)](https://sciwheel.com/work/citation?ids=11552465&pre=&suf=&sa=0&dbf=0). Herein, the variable used for race and ethnicity can be considered an ethnoracial variable, combining the two constructs [(8)](https://sciwheel.com/work/citation?ids=13920047&pre=&suf=&sa=0&dbf=0).

Season of conception has been linked to PTB and seasonal changes in the physical environment (e.g., heat, rainfall and flooding, exposure to sunlight) are known to influence contaminant levels in well water or volume of drinking water consumed [(9,10)](https://sciwheel.com/work/citation?ids=14428848,12998144&pre=&pre=&suf=&suf=&sa=0,0&dbf=0&dbf=0). Season of conception was derived from reported gestational age and date of birth on birth certificates and coded as disjoint indicator variables (winter, spring, summer, and fall). To capture co-pollutants as much as possible, nitrate and nitrite concentrations also measured in well water test reports in the NC-WELL database were averaged at a census tract level as a proxy for general agricultural contamination, which likely co-occurs with metals in well water and has been linked to PTB [(11)](https://sciwheel.com/work/citation?ids=14428912&pre=&suf=&sa=0&dbf=0). Concentrations for nitrates and nitrites reported below the LOR were imputed as LOR/$\sqrt{2}$. The average of the nitrates and nitrites concentration was then coded as less than or equal to the 50^th^ percentile, above the 50^th^ percentile and less than or equal to the 90^th^ percentile, or above the 90^th^ percentile, as an ordinal categorical variable.

***Metal mixtures modeling.*** Under additivity of the metals, each metal contributes to the overall effect in negative or positive direction, and each exposure has an associated weight corresponding to its proportion of the negative or positive “partial effect.” Independent effect sizes are calculated as the product of the weight and the partial effect of interest. For instance, a component of the mixture may have a weight of -1, indicating that it is the only contributor in the negative direction to the overall effect; however, its independent effect may be small compared to other components, indicating the strength of its association with the outcome of interest is minimal.

While the partial effects derived from the weights indicate the effect of component metals in each direction, they are not valid for inference because they depend on model estimates. We used sample splitting to estimate partial effects within the quantile-based g-computation R package to address this shortcoming, specifically using the *qgcomp.partials* function. In this process, the sample is split into a training (30%) and a validation (70%) set with random allocation. In the training dataset, a quantile-based g-computation model was fit to assess whether a metal was considered to have a positive or negative weight. Then, utilizing the validation set, quantile-based g-computation is used to estimate the effect of two distinct metal mixtures while adjusting for other metals: metals found in the training set to have a positive (or negative) effect in the metal mixture. As a sensitivity analysis to evaluate the effect of sample splitting on the stability of the partial effects modeling results, this process was repeated 12 times randomly splitting the dataset into training and validation sets each time. This number was chosen to balance having sufficient repeated cycles and data interpretation.

***Sensitivity analysis.*** To refine exposure assessment, we repeated the single metals modeling and mixtures modeling steps on a subsetted cohort more likely to be using private well water as their primary drinking water source. We generated a dataset of the number of predicted private well users in a census tract based on Johnson et al. 2019 [(12)](https://sciwheel.com/work/citation?ids=9562153&pre=&suf=&sa=0&dbf=0). The raster data published in Johnson et al. 2019 was converted into tabular format utilizing the zonal statistics tool in ArcGIS (v10.8.1). Specifically, the zone layer was census tract boundaries from 2010, the value layer was the raster data describing the number of private well users in a 1km x1km grid, and the output data was the number of private well users in a census tract. Using the estimated population based on the 2010 census in each census tract, obtained from ArcGIS, the percentage of private well water users in a tract was calculated. Two different nested cohorts were generated, one restricted to only birth certificate records where maternal residence at delivery was in a census tract with 50% or more predicted well water users (n=202,897, 15.3% of the study population), and one restricted to census tracts with 25% or more predicted well water users (n=445,002, 33.5%).

**Supplemental Information for Results**

***Effect of metal mixtures in private wells on the odds of preterm birth.*** The partial effects modeling was repeated 12 times to evaluate the influence of the random sample splitting on the results. Sample splitting generally demonstrated little influence over the results (**Table S3, S4**). Specifically, in 75% of the cycles, for metals positively associated with preterm birth in the training set, the mixture effect derived in the validation set demonstrated an increased risk with an >1 OR and confidence intervals that did not span the null, mirroring the results presented in the main analysis (**Table S3)**. In all but one of the cycles, metals negatively associated with preterm birth in the training set demonstrated a null mixture effect in the validation set (**Table S3)**. Across cycles, arsenic and zinc were consistently found to be negatively associated with preterm birth in the training set and contributed negatively to the effect in the validation set (**Table S4**). Conversely, cadmium was consistently found to be positively associated with preterm birth in the training set and to contribute positively to the effect in the validation set (**Table S4**). Lead was mostly found to be positively associated with preterm birth in the training set; in the two cycles where it was classified as being among the negative direction metals, its contribution to the metal mixture was still positive (**Table S4**). Manganese, copper, and chromium were inconsistent in the direction of their effect across the repeated cycles (**Table S4**).

***Sensitivity analysis.*** As a sensitivity analysis, the models were run on two subsets of the data: one restricted to only birth certificate records where residence at delivery was in a census tract with 50% or more predicted well water users and one restricted to census tracts with 25% or more predicted well water users. In the single metals analysis, the trends for most metals in terms of the directionality of the relationship with preterm birth were similar in both subsetted cohorts to the full cohort (**Table S6**). However, in the subsetted cohorts, most ORs estimated spanned the null. In the upper exposure category of cadmium (>=90^th^ percentile), unlike in the complete study population analysis, the OR for comparison to the lowest exposure category (<=50^th^ percentile) in both nested cohorts was <1 (**Table S6**). Additionally, in the upper exposure category of copper, also unlike in the complete study population analysis, the OR for comparison to the lowest exposure category (<=50^th^ percentile) in both nested cohorts was >1 (**Table S6**).

In the metal mixtures analysis, similar results in the subsetted data were obtained as calculated in the full cohort; however, all mixture effect estimates had confidence intervals that spanned the null, including in the partial effects modeling results (**Table S7**). In both subsetted cohorts, lead, chromium, and cadmium had positive weights in the standard quantile-based g-computation, indicating their contribution to increasing the risk of preterm birth, mirroring findings from the full cohort. Also, in both cohorts, in the standard and partial effects modeling, zinc and arsenic displayed consistent negative weighting indicating their contribution to a reduction in the risk of preterm birth, matching the findings from the complete cohort analysis.

**Figure S1.**  Flow diagram demonstrating the construction of the retrospective cohort used in the analysis, derived from live births between 2003 to 2015* in North Carolina.

**Figure S2.**  Directed Acyclic Graph describing relationship between well water metal concentrations, preterm birth, and critical covariates

**Table S1**. The EPA Maximum Contaminant Level Goal (MCLG) and EPA regulatory standard used in the study and well as the number and percentage of well water tests above and below limit of reporting for each metal. Note that these numbers are derived from the pre-imputation metals data. Following imputation, there were values for all n=117,798 well water metal tests included, ie. no missing data remaining.

|  |  |  |  | **Non missing** | | **Missing** |
| --- | --- | --- | --- | --- | --- | --- |
|  | **EPA MCLG** | **EPA regulatory standard^1^** | **LOR value** | **> LOR** | **<= LOR** |  |
|  | ppb | ppb | ppb | n (%) | n (%) | n (%) |
| Arsenic | 0 | 10 | 5 | 7198 (6.52) | 103184 (93.48) | 7416 (6.30) |
| Cadmium | 5 | 5 | 1 | 255 (0.34) | 75137 (99.66) | 42406 (36.00) |
| Chromium | 100 | 100 | 10 | 996 (1.52) | 64454 (98.48) | 52348 (44.44) |
| Copper | 1300 | 1300 | 50 | 7174 (10.93) | 58445 (89.07) | 52179 (44.30) |
| Lead | 0 | 15 | 5 | 8865 (8.04) | 101382 (91.96) | 7551 (6.41) |
| Manganese | n/a* | 300 | 8 | 33648 (30.54) | 76527 (69.46) | 7623 (6.47) |
| Zinc | n/a* | 5000 | 5 | 21968 (33.66) | 43296 (66.34) | 52534 (44.60) |
| Nitrites | 10000 | 10000** | 100 | 513 (1.11) | 45518 (98.89) | 71767 (60.92) |
| Nitrates | 1000 | 1000** | 1000 | 10196 (22.15) | 35837 (77.85) | 71765 (60.92) |

^1^This column lists the regulatory standard utilized in this study. For arsenic, cadmium and chromium, the Maximum Contaminant Level (MCL) was utilized. For copper and lead, the Treatment Technique (TT) level was utilized. For manganese, the EPA Health Advisory Level was utilized (note that there is also a secondary MCL of 50ppb, however this is based more on odor and taste than the health effects of chronic exposure). For zinc, the secondary MCL was utilized.

*No MCLG exists

**We did not utilize the MCLs for nitrates or nitrites in this study.

**Table S2.** Summary of the results from the 12 repeated cycles of partial effects quantile-based g-computation. Each cycle was run using a different random number to select the training and validation datasets (still set at 30%, 70% of the overall study population). The table details the mixture effect ORs for the metals that were in the negative direction in the training set (Direction column= negative) and metals that were in the positive direction in the training set (Direction column= positive)

| **Cycle** | **Direction**  *(ie. mixture matrix includes all metals with an effect in this direction in the training set)* | **OR (95% CI)** |
| --- | --- | --- |
| 1 | positive | 1.02 (1.00,1.03) |
| 1 | negative | 0.99 (0.98,1.01) |
| 2 | positive | 1.01 (1.00,1.03) |
| 2 | negative | 1.00 (0.99,1.02) |
| 3 | positive | 1.02 (1.00,1.03) |
| 3 | negative | 0.99 (0.98,1.01) |
| 4 | positive | 1.01 (0.99,1.02) |
| 4 | negative | 1.01 (1.00,1.03) |
| 5 | positive | 1.02 (1.01,1.03) |
| 5 | negative | 1.00 (0.98,1.01) |
| 6 | positive | 1.01 (1.00,1.02) |
| 6 | negative | 0.99 (0.98,1.01) |
| 7 | positive | 1.01 (0.99,1.02) |
| 7 | negative | 1.00 (0.98,1.01) |
| 8 | positive | 1.02 (1.01,1.03) |
| 8 | negative | 0.99 (0.98,1.01) |
| 9 | positive | 1.01 (1.00,1.03) |
| 9 | negative | 1.00 (0.98,1.01) |
| 10 | positive | 1.02 (1.01,1.03) |
| 10 | negative | 0.99 (0.98,1.00) |
| 11 | positive | 1.01 (1.00,1.03) |
| 11 | negative | 0.99 (0.98,1.00) |
| 12 | positive | 1.03 (1.01,1.04) |
| 12 | negative | 1.00 (0.99,1.01) |

**Table S3.** Summary of the results from the 12 repeated cycles of partial effects quantile-based g-computation. Each cycle was run using a different random number to select the training and validation datasets (still set at 30%, 70% of the overall study population). The table details, for each cycle, the direction of each metal in the training set and the weight the metal ultimately was assigned as a component of the mixture effect calculated in the validation set.

| **Cycle** | **Metal** | **Direction of metal in training set** | **Weight in validation set model** |
| --- | --- | --- | --- |
| 1 | Arsenic | negative | -0.66 |
| 2 | Arsenic | negative | -0.50 |
| 3 | Arsenic | negative | -0.71 |
| 4 | Arsenic | negative | -0.38 |
| 5 | Arsenic | negative | -0.65 |
| 6 | Arsenic | negative | -0.70 |
| 7 | Arsenic | negative | -0.31 |
| 8 | Arsenic | negative | -0.52 |
| 9 | Arsenic | negative | -0.44 |
| 10 | Arsenic | negative | -0.37 |
| 11 | Arsenic | negative | -0.69 |
| 12 | Arsenic | negative | -0.82 |
| 1 | Cadmium | positive | 0.68 |
| 2 | Cadmium | positive | 0.75 |
| 3 | Cadmium | positive | 0.43 |
| 4 | Cadmium | positive | 1.00 |
| 5 | Cadmium | positive | 0.55 |
| 6 | Cadmium | positive | 0.82 |
| 7 | Cadmium | positive | 0.65 |
| 8 | Cadmium | positive | 0.47 |
| 9 | Cadmium | positive | 0.63 |
| 10 | Cadmium | positive | 0.75 |
| 11 | Cadmium | positive | 0.52 |
| 12 | Cadmium | positive | 0.63 |
| 1 | Chromium | positive | -0.03 |
| 2 | Chromium | positive | 0.25 |
| 3 | Chromium | positive | 0.17 |
| 4 | Chromium | negative | 0.22 |
| 5 | Chromium | positive | 0.23 |
| 6 | Chromium | negative | 1.00 |
| 7 | Chromium | positive | 0.12 |
| 8 | Chromium | positive | 0.12 |
| 9 | Chromium | negative | 0.60 |
| 10 | Chromium | positive | -1.00 |
| 11 | Chromium | positive | 0.14 |
| 12 | Chromium | positive | 0.11 |
| 1 | Copper | negative | 1.00 |
| 2 | Copper | positive | -0.43 |
| 3 | Copper | negative | 0.30 |
| 4 | Copper | positive | -0.57 |
| 5 | Copper | negative | 0.82 |
| 6 | Copper | negative | -0.09 |
| 7 | Copper | positive | -0.65 |
| 8 | Copper | negative | 0.29 |
| 9 | Copper | negative | 0.40 |
| 10 | Copper | negative | 0.11 |
| 11 | Copper | positive | -0.59 |
| 12 | Copper | negative | 0.83 |
| 1 | Lead | positive | 0.32 |
| 2 | Lead | negative | 1.00 |
| 3 | Lead | positive | 0.40 |
| 4 | Lead | negative | 0.78 |
| 5 | Lead | positive | 0.23 |
| 6 | Lead | positive | 0.18 |
| 7 | Lead | positive | 0.23 |
| 8 | Lead | positive | 0.41 |
| 9 | Lead | positive | 0.37 |
| 10 | Lead | positive | 0.25 |
| 11 | Lead | positive | 0.34 |
| 12 | Lead | positive | 0.26 |
| 1 | Manganese | positive | -0.97 |
| 2 | Manganese | positive | -0.57 |
| 3 | Manganese | negative | 0.70 |
| 4 | Manganese | positive | -0.43 |
| 5 | Manganese | negative | 0.18 |
| 6 | Manganese | negative | -0.01 |
| 7 | Manganese | positive | -0.35 |
| 8 | Manganese | negative | 0.71 |
| 9 | Manganese | positive | -1.00 |
| 10 | Manganese | negative | 0.89 |
| 11 | Manganese | positive | -0.41 |
| 12 | Manganese | negative | 0.17 |
| 1 | Zinc | negative | -0.34 |
| 2 | Zinc | negative | -0.50 |
| 3 | Zinc | negative | -0.29 |
| 4 | Zinc | negative | -0.62 |
| 5 | Zinc | negative | -0.35 |
| 6 | Zinc | negative | -0.20 |
| 7 | Zinc | negative | -0.69 |
| 8 | Zinc | negative | -0.48 |
| 9 | Zinc | negative | -0.56 |
| 10 | Zinc | negative | -0.63 |
| 11 | Zinc | negative | -0.31 |
| 12 | Zinc | negative | -0.18 |

**Table S4.** Crude and adjusted odds ratios for the odds of (A) very preterm birth and (B) extremely preterm birth, comparing individuals in tracts with varying concentrations of individual metal concentrations reported in private wells. Note that for each metal, two models were fit. First, models were fit comparing individuals in tracts with low (mean tract-level metal concentration =<50^th^ percentile of state-wide metal concentration), medium (mean tract-level metal concentration >50^th^ and <90th percentile of state-wide metal concentration) and high (mean tract-level metal concentration >= 90th percentile of state-wide metal concentration) levels. Second, models were fit comparing individuals in tracts in which at least 25% or more well water tests reported concentrations above the EPA standard.

|  | **ppb** | | **Non-cases** | **Cases** | **Crude OR (95% CI)** | | | **Adjusted* OR (95% CI)** | |
| --- | --- | --- | --- | --- | --- | --- | --- | --- | --- |
| 1. ***Very preterm birth*** | | | | | | | | | |
| **Arsenic** |  | |  |  |  |  | | |  |
| <=50^th^ perc | | 0.220 | 660497 | 4862 | 1.00 (ref.) | 1.00 (ref.) | | | |
| >50^th^ perc to <90th perc | |  | 527844 | 3733 | 0.96 (0.92,1.00) | 0.98 (0.93,1.04) | | | |
| >=90th perc | | 2.838 | 131235 | 900 | 0.93 (0.87,1.00) | 0.956 (0.88,1.04) | | | |
|  |  | |  |  |  |  | | |  |
| tract with <25% of tests >=EPA limit | | limit = 10 | 1296545 | 9340 | 1.00 (ref.) | 1.00 (ref.) | | | |
| tract with >=25% of tests >=EPA limit | |  | 23031 | 155 | 0.93 (0.79,1.09) | 0.90 (0.75,1.06) | | | |
|  |  | |  |  |  |  | | |  |
| **Cadmium** |  | |  |  |  |  | | |  |
| <=50^th^ perc | | 0.007 | 659740 | 4876 | 1.00 (ref.) | 1.00 (ref.) | | | |
| >50^th^ perc to <90th perc | |  | 527967 | 3748 | 0.978 (0.94,1.02) | 1.01 (0.96,1.07) | | | |
| >=90th perc | | 0.127 | 131869 | 871 | 1.06 (0.99,1.14) | 1.06 (0.97,1.15) | | | |
|  |  | |  |  |  |  | | |  |
| tract with <25% of tests >=EPA limit | | Limit = 5 | 1314329 | 9457 | 1.00 (ref.) | 1.00 (ref.) | | | |
| tract with >=25% of tests >=EPA limit | |  | 5247 | 38 | 1.01 (0.72,1.36) | 1.08 (0.76,1.50) | | | |
|  |  | |  |  |  |  | | |  |
| **Chromium** |  | |  |  |  |  | | |  |
| <=50^th^ perc | 0.579 | | 659702 | 4867 | 1.00 (ref.) | 1.00 (ref.) | | | |
| >50^th^ perc to <90th perc |  | | 528214 | 3733 | 0.96 (0.92,1.00) | 1.02 (0.97,1.08) | | | |
| >=90th perc | 2.872 | | 131660 | 895 | 0.92 (0.86,0.99) | 0.96 (0.88,1.05) | | | |
|  |  | |  |  |  |  | | | |
| tract with <25% of tests >=EPA limit | Limit= 100 | | 1319097 | 479 | 1.00 (ref.) | 1.00 (ref.) | | | |
| tract with >=25% of tests >=EPA limit |  | | 9490 | 5 | 1.45 (0.52,3.14) | 0.98 (0.30,2.30) | | | |
|  |  | |  |  |  |  | | |  |
| **Copper** |  | |  |  |  |  | | | |
| <=50^th^ perc | 21.096 | | 659613 | 5008 | 1.00 (ref.) | 1.00 (ref.) | | | |
| >50^th^ perc to <90th perc |  | | 528285 | 3595 | 0.90 (0.86,0.94) | 0.95 (0.90,1.00) | | | |
| >=90th perc | 141.545 | | 131678 | 892 | 0.89 (0.83,0.96) | 0.92 (0.84,1.01) | | | |
|  |  | |  |  |  |  | | | |
| tract with <25% of tests >=EPA limit | Limit= 1300 | | 1314893 | 9457 | 1.00 (ref.) | 1.00 (ref.) | | | |
| tract with >=25% of tests >=EPA limit |  | | 4683 | 38 | 1.13 (0.81,1.53) | n/a | | | |
|  |  | |  |  |  |  | | |  |
| **Lead** |  | |  |  |  |  | | |  |
| <=50^th^ perc | | 1.433 | 659917 | 4755 | 1.00 (ref.) | 1.00 (ref.) | | | |
| >50^th^ perc to <90th perc | |  | 527881 | 3763 | 0.99 (0.95,1.03) | 1.01 (0.96,1.06) | | | |
| >=90th perc | | 9.104 | 131778 | 977 | 1.03 (0.96,1.10) | 1.02 (0.94,1.12) | | | |
|  |  | |  |  |  |  | | |  |
| tract with <25% of tests >=EPA limit | | Limit=15 | 1289608 | 9256 | 1.00 (ref.) | 1.00 (ref.) | | | |
| tract with >=25% of tests >=EPA limit | |  | 29968 | 239 | 1.11 (0.97,1.26) | 1.10 (0.87,1.38) | | | |
|  |  | |  |  |  |  | | |  |
| **Manganese** |  | |  |  |  |  | | |  |
| <=50^th^ perc | | 33.626 | 660098 | 4828 | 1.00 (ref.) | 1.00 (ref.) | | | |
| >50^th^ perc to <90th perc | |  | 528005 | 3813 | 0.99 (0.95,1.03) | 1.00 (0.95,1.05) | | | |
| >=90th perc | | 188.923 | 131473 | 854 | 0.89 (0.83,0.96) | 0.93 (0.85,1.01) | | | |
|  |  | |  |  |  |  | | |  |
| tract with <25% of tests >=EPA limit | | Limit=300 | 1273681 | 9197 | 1.00 (ref.) | 1.00 (ref.) | | | |
| tract with >=25% of tests >=EPA limit | |  | 45895 | 298 | 0.90 (0.80,1.01) | 0.912 (0.78,1.06) | | | |
|  |  | |  |  |  |  | | |  |
| **Zinc** |  | |  |  |  |  | | |  |
| <=50^th^ perc | | 126.764 | 659797 | 4888 | 1.00 (ref.) | 1.00 (ref.) | | | |
| >50^th^ perc to <90th perc | |  | 527796 | 3690 | 0.94 (0.90,0.99) | 0.99 (0.94,1.04) | | | |
| >=90th perc | | 1592.883 | 131983 | 917 | 0.94 (0.87,1.01) | 0.97 (0.89,1.06) | | | |
|  |  | |  |  |  |  | | |  |
| tract with <25% of tests >=EPA limit | | Limit= 5000 | 1309882 | 9435 | 1.00 (ref.) | 1.00 (ref.) | | | |
| tract with >=25% of tests >=EPA limit | |  | 9694 | 60 | 0.86 (0.66,1.10) | 0.38 (0.06,1.18) | | | |
|  | |  |  |  |  |  | | | |
| 1. ***Extremely preterm birth*** | | | | | | | | | |
| **Arsenic** | |  |  |  |  | |  | | |
| <=50^th^ perc | | 0.220 | 661415 | 3944 | 1.00 (ref.) | | 1.00 (ref.) | | |
| >50^th^ perc to <90th perc | |  | 528475 | 3102 | 0.98 (0.94,1.03) | | 1.04 (0.98,1.11) | | |
| >=90th perc | | 2.838 | 131463 | 672 | 0.86 (0.79,0.93) | | 0.92 (0.83,1.01) | | |
|  | |  |  |  |  | |  | | |
| tract with <25% of tests >=EPA limit | | limit = 10 | 1298295 | 7590 | 1.00 (ref.) | | 1.00 (ref.) | | |
| tract with >=25% of tests >=EPA limit | |  | 23058 | 128 | 0.95 (0.79,1.13) | | 0.89 (0.73,1.08) | | |
|  | |  |  |  |  | |  | | |
| **Cadmium** | |  |  |  |  | |  | | |
| <=50^th^ perc | | 0.007 | 660624 | 4006 | 1.00 (ref.) | | 1.00 (ref.) | | |
| >50^th^ perc to <90th perc | |  | 529000 | 2932 | 0.91 (0.87,0.96) | | 0.99 (0.93,1.05) | | |
| >=90th perc | | 0.127 | 131729 | 780 | 0.98 (0.90,1.05) | | 1.01 (0.92,1.11) | | |
|  | |  |  |  |  | |  | | |
| tract with <25% of tests >=EPA limit | | Limit = 5 | 1316111 | 23058 | 1.00 (ref.) | | 1.00 (ref.) | | |
| tract with >=25% of tests >=EPA limit | |  | 7590 | 128 | 1.41 (1.03,1.87) | | 1.56 (1.12,2.10) | | |
|  | |  |  |  |  | |  | | |
| **Chromium** | |  |  |  |  | |  | | |
| <=50^th^ perc | | 0.579 | 660428 | 4141 | 1.00 (ref.) | | 1.00 (ref.) | | |
| >50^th^ perc to <90th perc | |  | 529052 | 2895 | 0.87 (0.83,0.92) | | 0.94 (0.88,1.00) | | |
| >=90th perc | | 2.872 | 131873 | 682 | 0.83 (0.76,0.89) | | 0.87 (0.79,0.96) | | |
|  | |  |  |  |  | |  | | |
| tract with <25% of tests >=EPA limit | | Limit= 100 | 1320871 | 7716 | 1.00 (ref.) | | 1.00 (ref.) | | |
| tract with >=25% of tests >=EPA limit | |  | 482 | 2 | 0.71 (0.12,2.20) | | 0.53 (0.09,1.66) | | |
|  | |  |  |  |  | |  | | |
| **Copper** | |  |  |  |  | |  | | |
| <=50^th^ perc | | 21.096 | 660510 | 4111 | 1.00 (ref.) | | 1.00 (ref.) | | |
| >50^th^ perc to <90th perc | |  | 529037 | 2843 | 0.86 (0.82,0.91) | | 0.97 (0.91,1.03) | | |
| >=90th perc | | 141.545 | 131803 | 764 | 0.93 (0.86,1.01) | | 0.91 (0.82,1.01) | | |
|  | |  |  |  |  | |  | | |
| tract with <25% of tests >=EPA limit | | Limit= 1300 | 1316666 | 4687 | 1.00 (ref.) | | 1.00 (ref.) | | |
| tract with >=25% of tests >=EPA limit | |  | 7684 | 34 | 1.24 (0.87,1.71) | | n/a | | |
|  | |  |  |  |  | |  | | |
| **Lead** | |  |  |  |  | |  | | |
| <=50^th^ perc | | 1.433 | 660703 | 3969 | 1.00 (ref.) | | 1.00 (ref.) | | |
| >50^th^ perc to <90th perc | |  | 528719 | 2925 | 0.92 (0.88,0.97) | | 0.97 (0.92,1.03) | | |
| >=90th perc | | 9.104 | 131931 | 824 | 1.04 (0.96,1.12) | | 1.06 (0.96,1.16) | | |
|  | |  |  |  |  | |  | | |
| tract with <25% of tests >=EPA limit | | Limit=15 | 1316111 | 7675 | 1.00 (ref.) | | 1.00 (ref.) | | |
| tract with >=25% of tests >=EPA limit | |  | 5242 | 43 | 1.36 (1.19,1.54) | | 1.46 (1.17,1.81) | | |
|  | |  |  |  |  | |  | | |
| **Manganese** | |  |  |  |  | |  | | |
| <=50^th^ perc | | 33.626 | 660937 | 3989 | 1.00 (ref.) | | 1.00 (ref.) | | |
| >50^th^ perc to <90th perc | |  | 528825 | 2983 | 0.94 (0.89,0.98) | | 0.98 (0.93,1.04) | | |
| >=90th perc | | 188.923 | 131581 | 746 | 0.94 (0.87,1.02) | | 1.02 (0.92,1.12) | | |
|  | |  |  |  |  | |  | | |
| tract with <25% of tests >=EPA limit | | Limit=300 | 1275432 | 7446 | 1.00 (ref.) | | 1.00 (ref.) | | |
| tract with >=25% of tests >=EPA limit | |  | 45921 | 272 | 1.02 (0.90,1.14) | | 1.04 (0.88,1.21) | | |
|  | |  |  |  |  | |  | | |
| **Zinc** | |  |  |  |  | |  | | |
| <=50^th^ perc | | 126.764 | 660628 | 4057 | 1.00 (ref.) | | 1.00 (ref.) | | |
| >50^th^ perc to <90th perc | |  | 528546 | 2940 | 0.91 (0.86,0.95) | | 0.98 (0.92,1.04) | | |
| >=90th perc | | 1592.883 | 132179 | 721 | 0.89 (0.82,0.96) | | 0.88 (0.79,0.97) | | |
|  | |  |  |  |  | |  | | |
| tract with <25% of tests >=EPA limit | | Limit= 5000 | 1311666 | 9687 | 1.00 (ref.) | | 1.00 (ref.) | | |
| tract with >=25% of tests >=EPA limit | |  | 7651 | 67 | 1.19 (0.92,1.50) | | 0.68 (0.17,1.77) | | |

*adjusted for smoking, age, race/ethnicity, education, season of conception, tract-level poverty, tract-level nitrates and nitrites

Note that adjusted models for comparing individuals in tracts in which at least 25% or more well water tests reported concentrations above the EPA standard for copper did not converge.

**Table S5.** Summary of results from quantile-based g-computation modeling for very preterm birth and extremely preterm birth outcomes. Model A includes all metals in the exposure matrix. Models B and C contain metals that were associated in the positive direction and the negative direction, respectively, in the training data set in the quantile-based g computation partial effect modelling. The weights that sum to 1 for each of the adjusted models are also given.

| **Model** | **Interpretation** | **Crude OR (95% CI)** | **Adjusted* OR (95% CI)** | **Adjusted model weights** |
| --- | --- | --- | --- | --- |
| 1. ***Very preterm birth*** | | | | |
| 1. Standard quantile-based g-computation | Increasing all metals by one quartile (ppb) | 0.93 (0.90,0.96) | 0.98 (0.94,1.02) | \| Lead \| 0.62 \| \| --- \| --- \| \| Cadmium \| 0.30 \| \| Chromium \| 0.08 \| \| Copper \| -0.72 \| \| Arsenic \| -0.15 \| \| Manganese \| -0.09 \| \| Zinc \| -0.03 \| |
| 1. Positive direction partial effects quantile-based g-computation | Increasing all metals that were in the positive direction in the training set by one quartile (ppb) | 0.97 (0.95,1.00) | 0.98 (0.94,1.03) | \| Lead \| 1 \| \| --- \| --- \| \| Zinc \| -0.68 \| \| Manganese \| -0.32 \| |
| 1. Negative direction partial effects quantile-based g-computation | Increasing all metals that were in the negative direction in the training set by one quartile (ppb) | 0.91 (0.88,0.95) | 0.99 (0.94,1.04) | \| Cadmium \| 0.70 \| \| --- \| --- \| \| Chromium \| 0.30 \| \| Copper \| -0.87 \| \| Arsenic \| -0.13 \| |
| 1. ***Extremely preterm birth*** | | | | |
| 1. Standard quantile-based g-computation | Increasing all metals by one quartile (ppb) | 0.88 (0.85,0.91) | 0.97 (0.93,1.02) | \| Lead \| 0.38 \| \| --- \| --- \| \| Arsenic \| 0.34 \| \| Cadmium \| 0.18 \| \| Manganese \| 0.10 \| \| Copper \| -0.37 \| \| Chromium \| -0.32 \| \| Zinc \| -0.31 \| |
| 1. Positive direction partial effects quantile-based g-computation | Increasing all metals that were in the positive direction in the training set by one quartile (ppb) | 0.99 (0.96,1.01) | 0.97 (0.92,1.02) | \| Arsenic \| 0.74 \| \| --- \| --- \| \| Lead \| 0.26 \| \| Chromium \| -0.96 \| \| Cadmium \| -0.04 \| |
| 1. Negative direction partial effects quantile-based g-computation | Increasing all metals that were in the negative direction in the training set by one quartile (ppb) | 0.87 (0.83,0.90) | 0.96 (0.92,1.01) | \| Manganese \| 1 \| \| --- \| --- \| \| Zinc \| -0.53 \| \| Copper \| -0.47 \| |

*adjusted for smoking, age, race/ethnicity, education, season of conception, tract-level poverty, tract-level nitrates and nitrites

**Table S6**. Adjusted odds ratios for the odds of preterm birth, comparing individuals in tracts with varying concentrations of individual metal concentrations reported in private wells. Models were fit comparing individuals in tracts with low (mean tract-level metal concentration =<50^th^ percentile of state-wide metal concentration), medium (mean tract-level metal concentration >50^th^ and <90^th^ percentile of state-wide metal concentration) and high (mean tract-level metal concentration >= 90^th^ percentile of state-wide metal concentration) levels. Model results are presented for two nested cohorts: first, individuals residing in a tract estimated to have over 25% of residents on private well water; second individuals residing in a tract estimated to have over 50% of residents on private well water.

|  | | **ppb** | **Population residing in tract with over 25% of residents estimated to be private well water users**  **Adjusted* OR (95% CI)** | **Population residing in tract with over 50% of residents estimated to be private well water users**  **Adjusted* OR (95% CI)** |
| --- | --- | --- | --- | --- |
| **Arsenic** |  |  |  |  |
| <=50^th^ perc | | 0.220 | 1.00 (ref.) | 1.00 (ref.) |
| >50^th^ perc to <90th perc | |  | 0.97 (0.95,0.99) | 0.98 (0.94,1.01) |
| >=90th perc | | 2.838 | 0.95 (0.91,0.98) | 0.91 (0.86,0.97) |
|  |  |  |  |  |
| **Cadmium** |  |  |  |  |
| <=50^th^ perc | | 0.007 | 1.00 (ref.) | 1.00 (ref.) |
| >50^th^ perc to <90th perc | |  | 1.00 (0.98,1.03) | 1.01 (0.98,1.05) |
| >=90th perc | | 0.127 | 0.95 (0.92,1.00) | 0.94 (0.88,1.01) |
|  |  |  |  |  |
| **Chromium** |  |  |  |  |
| <=50^th^ perc | | 0.579 | 1.00 (ref.) | 1.00 (ref.) |
| >50^th^ perc to <90th perc | |  | 1.03 (1.00,1.05) | 1.07 (1.04,1.11) |
| >=90th perc | | 2.872 | 1.04 (0.99,1.08) | 1.02 (0.95,1.09) |
|  |  |  |  |  |
| **Copper** |  |  |  |  |
| <=50^th^ perc | | 21.096 | 1.00 (ref.) | 1.00 (ref.) |
| >50^th^ perc to <90th perc | |  | 1.00 (0.97,1.02) | 0.98 (0.95,1.01) |
| >=90th perc | | 141.545 | 1.02 (0.98,1.07) | 1.15 (1.06,1.25) |
|  |  |  |  |  |
| **Lead** |  |  |  |  |
| <=50^th^ perc | | 1.433 | 1.00 (ref.) | 1.00 (ref.) |
| >50^th^ perc to <90th perc | |  | 1.01 (0.98,1.03) | 1.02 (0.99,1.06) |
| >=90th perc | | 9.104 | 1.00 (0.96,1.05) | 0.98 (0.92,1.04) |
|  |  |  |  |  |
| **Manganese** |  |  |  |  |
| <=50^th^ perc | | 33.626 | 1.00 (ref.) | 1.00 (ref.) |
| >50^th^ perc to <90th perc | |  | 1.00 (0.97,1.02) | 1.00 (0.97,1.04) |
| >=90th perc | | 188.923 | 0.94 (0.91,0.98) | 0.95 (0.90,1.00) |
|  |  |  |  |  |
| **Zinc** |  |  |  |  |
| <=50^th^ perc | | 126.764 | 1.00 (ref.) | 1.00 (ref.) |
| >50^th^ perc to <90th perc | |  | 0.99 (0.97,1.01) | 0.99 (0.95,1.02) |
| >=90th perc | | 1592.883 | 0.95 (0.91,0.99) | 1.00 (0.94,1.06) |

**Table S7.** Summary of results from adjusted quantile-based g-computation modeling among the (A) population residing in tracts with over 25% of residents estimated to be private well water users and (B) population residing in tracts with over 50% of residents estimated to be private well water users. Model A includes all metals in the exposure matrix. Models B and C contain metals that were associated in the positive direction and the negative direction, respectively, in the training data set in the quantile-based g computation partial effect modelling. The weights that sum to 1 for each of the adjusted models are also given.

| **Model** | **Interpretation** | **Adjusted* OR (95% CI)** | **Adjusted model weights** |
| --- | --- | --- | --- |
| *(A) Population residing in tracts with over 25% of residents estimated to be private well water users* | | | |
| 1. Standard quantile-based g-computation | Increasing all metals by one quartile (ppb) | 0.98 (1.00,0.96) | \| Chromium \| 0.69 \| \| --- \| --- \| \| Cadmium \| 0.26 \| \| Copper \| 0.03 \| \| Lead \| 0.03 \| \| Arsenic \| -0.44 \| \| Zinc \| -0.41 \| \| Manganese \| -0.15 \| |
| 1. Positive direction partial effects quantile-based g-computation | Increasing all metals that were in the positive direction in the training set by one quartile (ppb) | 1.00 (1.02,0.98) | \| Chromium \| 0.80 \| \| --- \| --- \| \| Cadmium \| 0.20 \| \| Manganese \| -0.73 \| \| Copper \| -0.27 \| |
| 1. Negative direction partial effects quantile-based g-computation | Increasing all metals that were in the negative direction in the training set by one quartile (ppb) | 0.97 (0.99,0.95) | \| Lead \| 1.00 \| \| --- \| --- \| \| Arsenic \| -0.50 \| \| Zinc \| -0.50 \| |
| *(B) Population residing in tracts with over 50% of residents estimated to be private well water users* | | | |
| 1. Standard quantile-based g-computation | Increasing all metals by one quartile (ppb) | 1.00 (0.97,1.03) | \| Chromium \| 0.52 \| \| --- \| --- \| \| Cadmium \| 0.18 \| \| Lead \| 0.16 \| \| Copper \| 0.09 \| \| Manganese \| 0.05 \| \| Zinc \| -0.55 \| \| Arsenic \| -0.45 \| |
| 1. Positive direction partial effects quantile-based g-computation | Increasing all metals that were in the positive direction in the training set by one quartile (ppb) | 1.03 (0.99,1.06) | \| Chromium \| 0.65 \| \| --- \| --- \| \| Lead \| 0.25 \| \| Cadmium \| 0.11 \| \| Manganese \| -1.00 \| |
| 1. Negative direction partial effects quantile-based g-computation | Increasing all metals that were in the negative direction in the training set by one quartile (ppb) | 0.99 (0.96,1.02) | \| Copper \| 1.00 \| \| --- \| --- \| \| Zinc \| -0.79 \| \| Arsenic \| -0.21 \| |

**Bibliography**

[1.    Saccone G, Gragnano E, Ilardi B, Marrone V, Strina I, Venturella R, et al. Maternal and perinatal complications according to maternal age: A systematic review and meta-analysis. Int J Gynaecol Obstet. 2022 Oct;159(1):43–55.](https://sciwheel.com/work/bibliography/14433276)

[2.    Shah NR, Bracken MB. A systematic review and meta-analysis of prospective studies on the association between maternal cigarette smoking and preterm delivery. Am J Obstet Gynecol. 2000 Feb;182(2):465–72.](https://sciwheel.com/work/bibliography/8482414)

[3.    Blumenshine P, Egerter S, Barclay CJ, Cubbin C, Braveman PA. Socioeconomic disparities in adverse birth outcomes: a systematic review. Am J Prev Med. 2010 Sep;39(3):263–72.](https://sciwheel.com/work/bibliography/4216093)

[4.    Walker K, Herman M. tidycensus: Load US Census Boundary and Attribute Data as “tidyverse” and ’sf’-Ready Data Frames. https://CRAN.R-project.org/package=tidycensus; 2021.](https://sciwheel.com/work/bibliography/11789041)

[5.    Buescher PA, Gizlice Z, Jones-Vessey KA. Discrepancies between published data on racial classification and self-reported race: evidence from the 2002 North Carolina live birth records. Public Health Rep. 2005 Aug;120(4):393–8.](https://sciwheel.com/work/bibliography/7058715)

[6.    NVSS - Revisions of the U.S. Standard Certificates and Reports [Internet]. [cited 2023 Feb 22]. Available from: https://www.cdc.gov/nchs/nvss/revisions-of-the-us-standard-certificates-and-reports.htm](https://sciwheel.com/work/bibliography/14433251)

[7.    Flanagin A, Frey T, Christiansen SL, AMA Manual of Style Committee. Updated guidance on the reporting of race and ethnicity in medical and science journals. JAMA. 2021 Aug 17;326(7):621–7.](https://sciwheel.com/work/bibliography/11552465)

[8.    Martinez RAM, Andrabi N, Goodwin AN, Wilbur RE, Smith NR, Zivich PN. Conceptualization, Operationalization, and Utilization of Race and Ethnicity in Major Epidemiology Journals, 1995-2018: A Systematic Review. Am J Epidemiol. 2023 Feb 24;192(3):483–96.](https://sciwheel.com/work/bibliography/13920047)

[9.    Bodnar LM, Simhan HN. The prevalence of preterm birth and season of conception. Paediatr Perinat Epidemiol. 2008 Nov;22(6):538–45.](https://sciwheel.com/work/bibliography/14428848)

[10.   Rowles Iii LS, Hossain AI, Ramirez I, Durst NJ, Ward PM, Kirisits MJ, et al. Seasonal contamination of well-water in flood-prone colonias and other unincorporated U.S. communities. Sci Total Environ. 2020 Oct 20;740:140111.](https://sciwheel.com/work/bibliography/12998144)

[11.   Lin L, St Clair S, Gamble GD, Crowther CA, Dixon L, Bloomfield FH, et al. Nitrate contamination in drinking water and adverse reproductive and birth outcomes: a systematic review and meta-analysis. Sci Rep. 2023 Jan 11;13(1):563.](https://sciwheel.com/work/bibliography/14428912)

[12.   Johnson TD, Belitz K, Lombard MA. Estimating domestic well locations and populations served in the contiguous U.S. for years 2000 and 2010. Sci Total Environ. 2019 Oct 15;687:1261–73.](https://sciwheel.com/work/bibliography/9562153)
